# Probing the solution structure of the *E*. *coli* multidrug transporter MdfA using DEER distance measurements with nitroxide and Gd(III) spin labels

**DOI:** 10.1038/s41598-019-48694-0

**Published:** 2019-08-29

**Authors:** Eliane H. Yardeni, Thorsten Bahrenberg, Richard A. Stein, Smriti Mishra, Elia Zomot, Bim Graham, Kellie L. Tuck, Thomas Huber, Eitan Bibi, Hassane S. Mchaourab, Daniella Goldfarb

**Affiliations:** 1 Department of Biomolecular Sciences, Weizmann Institute of Science Rehovot, Rehovot, 76100 Israel; 2 Department of Chemical and Biological Physics, Weizmann Institute of Science, Rehovot, 76100 Israel; 3 Department of Molecular Physiology and Biophysics, Vanderbilt University, Nashville, TN USA; 4 Monash Institute of Pharmaceutical Sciences, Monash University, Parkville, VIC 3052 Australia; 5 School of Chemistry, Monash University, Wellington Road, Clayton, Victoria Australia; 6 Research School of Chemistry, The Australian National University, Canberra, ACT 2601 Australia

**Keywords:** Biophysical chemistry, Biophysics

## Abstract

Methodological and technological advances in EPR spectroscopy have enabled novel insight into the structural and dynamic aspects of integral membrane proteins. In addition to an extensive toolkit of EPR methods, multiple spin labels have been developed and utilized, among them Gd(III)-chelates which offer high sensitivity at high magnetic fields. Here, we applied a dual labeling approach, employing nitroxide and Gd(III) spin labels, in conjunction with Q-band and W-band double electron-electron resonance (DEER) measurements to characterize the solution structure of the detergent-solubilized multidrug transporter MdfA from *E*. *coli*. Our results identify highly flexible regions of MdfA, which may play an important role in its functional dynamics. Comparison of distance distribution of spin label pairs on the periplasm with those calculated using inward- and outward-facing crystal structures of MdfA, show that in detergent micelles, the protein adopts a predominantly outward-facing conformation, although more closed than the crystal structure. The cytoplasmic pairs suggest a small preference to the outward-facing crystal structure, with a somewhat more open conformation than the crystal structure. Parallel DEER measurements with the two types of labels led to similar distance distributions, demonstrating the feasibility of using W-band spectroscopy with a Gd(III) label for investigation of the structural dynamics of membrane proteins.

## Introduction

Distance measurement by DEER (double electron-electron resonance, also called PELDOR) is an established technique to track macromolecular structures^1^. It reports on the dipolar interaction between two electron spins, which can be converted into a distance due to its r^−3^ dependence, where r is the inter-spin distance. DEER measurements yield distance distributions, on the 1.5–8.0 nm scale, that can be extracted from the data using multiple approaches^2,3^. Two paramagnetic labels that are commonly, but not necessarily, of the same type are attached to proteins via site directed spin labeling (SDSL) to the thiol groups of native or genetically engineered cysteines^4^. The most commonly used spin label in DEER applications to proteins is MTSSL (S-(1-oxyl-2,2,5,5-tetramethyl-2,5-dihydro-1H-pyrrol-3-yl)methyl methanesulfonothioate). Its spectroscopic as well as biochemical advantages are well known and established^5–7^. However, there are instances when MTSSL is not the optimal spin label such as for in-cell applications where the paramagnetic nitroxide group is reduced to a diamagnetic hydroxyl amine^8^. Additionally, at high frequencies, like W-band (95 GHz), which are advantageous for high sensitivity^9^, data analysis of nitroxide DEER measurements can be complicated by orientation selection^10^. Therefore, alternatives to nitroxide spin labeling have been introduced in the past decade^11–13^, among them Gd(III) chelates, which are suitable for Q- and W-band frequencies (34 and 95 GHz, respectively) measurements^14–33^. Notably, at W-band they exhibit high sensitivity^34^ and the DEER traces are free of orientation selection effects^35^. Gd(III) is stable in reducing environments and so far most of the reported in-cell DEER measurements have employed Gd(III) labels^16,29,36–41^. Finally, having the choice of different spin labels allows for labeling strategies that afford measurement of three independent distances per sample as opposed to one distance provided by the standard two spin labels approach^42^.

DEER employing the MTSSL spin label has been applied to diverse membrane proteins with first applications appearing as early as 2004^43–45^ in various environments including detergents^46,47^, bicelles^48^, nanodiscs^49^, lipodiscs^50^, nanoparticles^50^, and cell membranes^51^, with emphasis on the conformational states underlying transport mechanisms^52–59^. The application of Gd(III) spin labels to membrane proteins have so far been limited to studying the oligomeric state of proteorhodopsin, showing that they are immune to multi-spin effects, which introduce uncertainties in the data analysis^24^. In addition, using a membrane embedded transmembrane model peptide^25^, it was shown that labeling the same two positions with Gd(III) spin labels or nitroxide spin labels yielded distance distributions different than those predicted, presumably because of their different interactions with the membrane owing to their different hydrophobicity. Therefore, employing DEER with different types of spin labels yields more detailed spatial information and possibly resolving context-dependent spin label effects.

**Figure 1:**
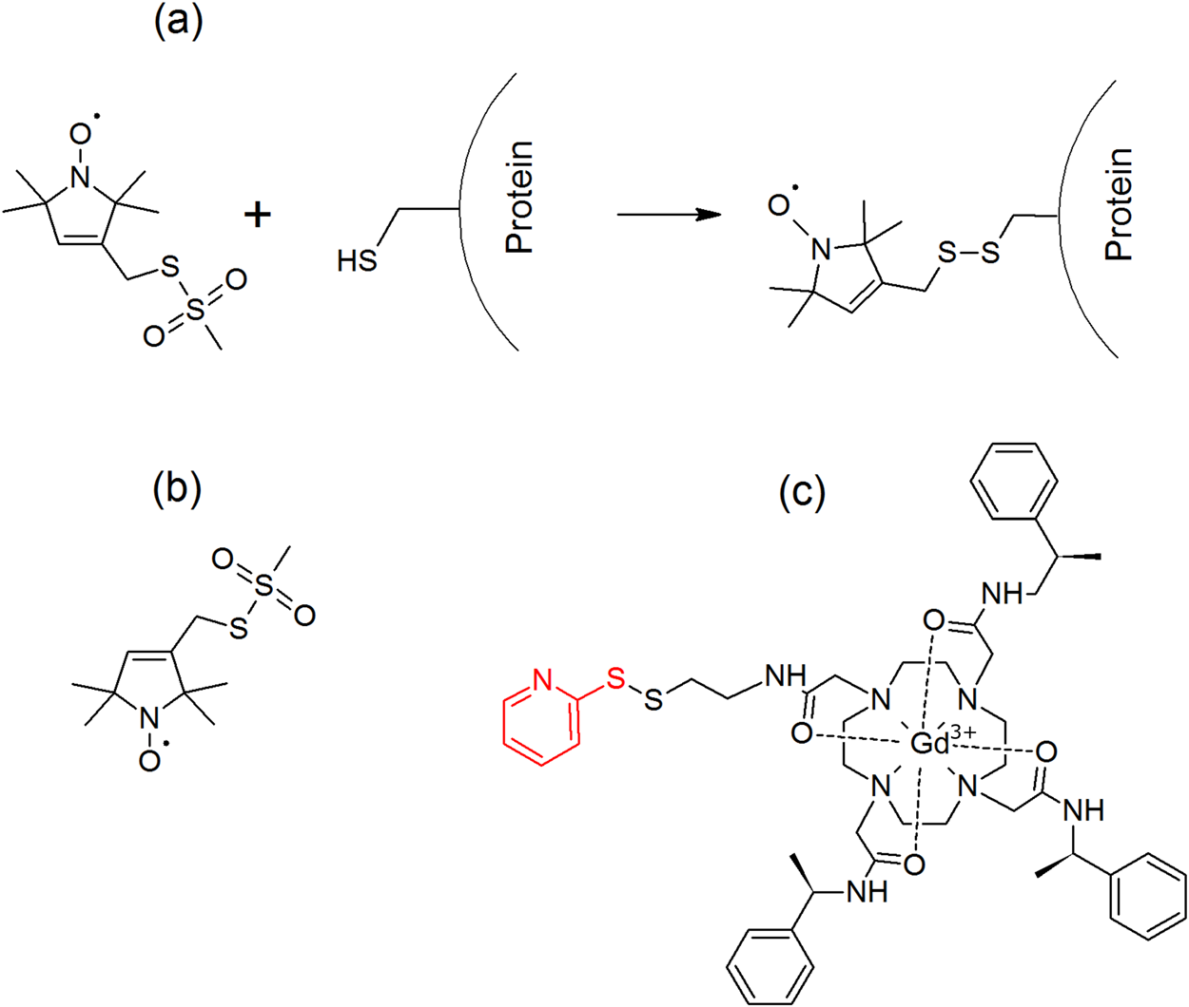
(**a**) Scheme of the labeling reaction of MTSSL with the thiol moiety of a protein’s cysteine; The chemical structure of (**b)** MTSSL and (**c**) C2-Gd. The leaving group in in the C2-Gd tag appears in red.

In this work we applied DEER with MTSSL, measured at Q-band, and with a Gd(III) spin label, Gd-C2 (Fig. 1) measured at W-band. Using these labels we characterized the conformational behavior of the membrane protein MdfA, an *Escherichia coli* secondary multidrug transporter (Mdr)^60^. In addition, the suitability of Gd(III) spin labels for the study of membrane proteins was explored. Multidrug transporters are membrane proteins that are able to export a variety of structurally dissimilar compounds out of cells^61^. They play a vital role in cellular protection against a variety of potential cytotoxic compounds, including antibiotics. The prototypic secondary Mdr transporter MdfA operates as substrate/H^+^ antiporter, importing one equivalent H^+^ in every export cycle^61^.

MdfA is composed of 410 amino acid residues, organized in 12 transmembrane (TM) helices, with both N- and C- termini located in the cytoplasm^62^. Previous studies have suggested that MdfA is unusually flexible^61^, which may explain why the wild type protein has never yielded highly diffracting crystals. A hypothetical 3D model for wild type MdfA^63^ has received direct support from the high-resolution crystal structure of an MdfA mutant, MdfA(Q131R) with a trapped ligand (pdb 4ZP0) in an inward facing structure (I_f_)^64^. This structure shows that, indeed, MdfA shares general structural characteristics of major facilitator superfamily (MFS) transporters^65^. These include the canonical MFS fold with two domains of six consecutive trans-membrane (TM) helices, termed the N- and C- domains, which exhibit a two-fold pseudosymmetry. More recently, a new structure of native MdfA bound to a FAB has been reported (pdb 6GV1) in an outward open conformation (O_o_)^66^. The current model posits that during its transport cycle MdfA undergoes a conformational cycle which involves both the I_f_ and O_o_ conformations, with the O_o_ conformation possibly capturing the state that releases substrates into the periplasm^67^. Following its protonation, MdfA probably assumes a ligand occluded I_f_ conformation. Thus, the two structures offer a partial view on the conformational behavior of MdfA. Further crystallization efforts as well as biochemical and spectroscopic studies are needed to reveal additional conformational states. Specifically, understanding the functional dynamics of MdfA in its natural environment requires accurate distance constraints between regions in the protein that undergo movement during substrate binding and release.

**Figure 2:**
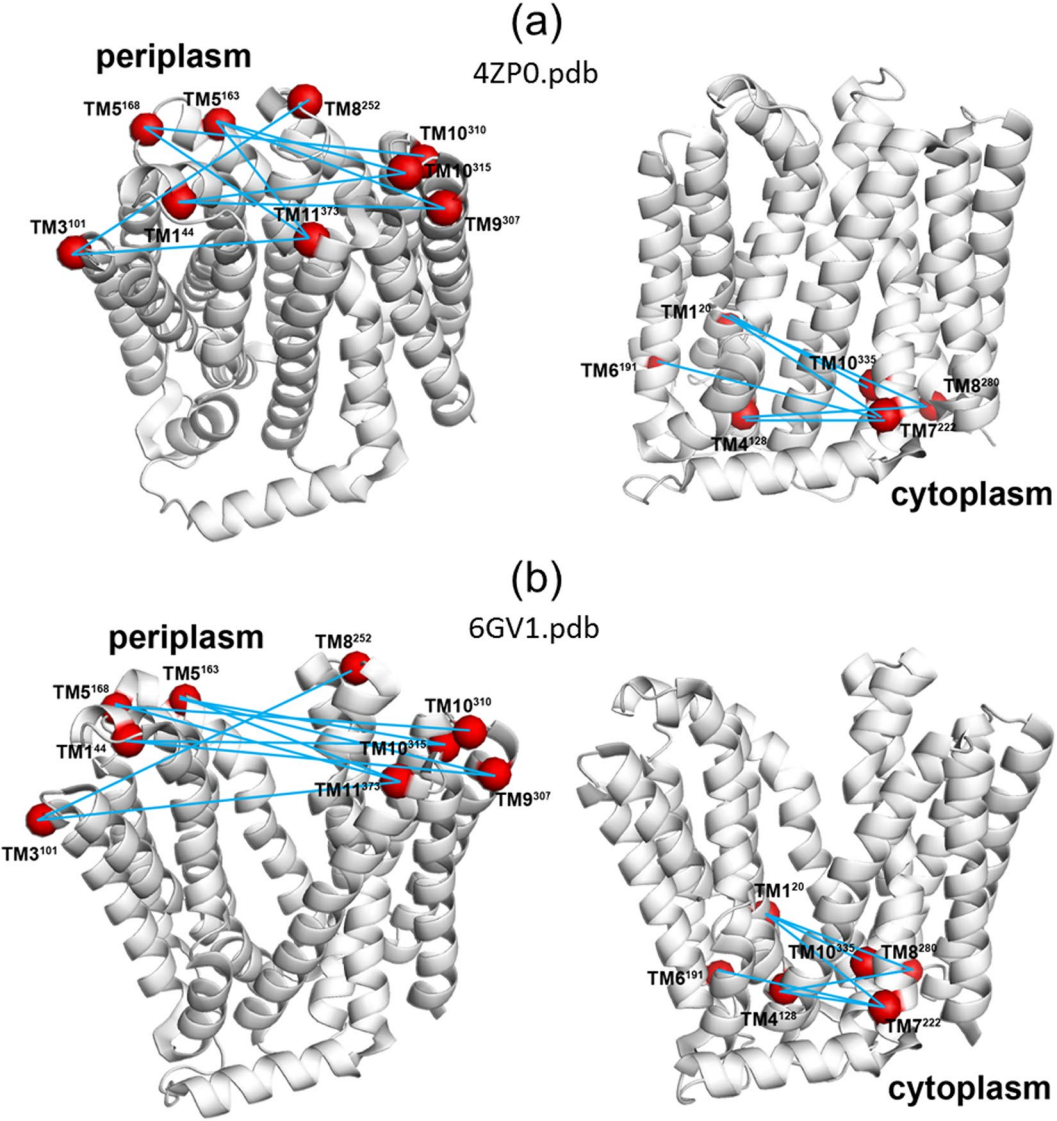
Crystal structures of MdfA with the locations of the spin labels in the periplasmic face (left) and cytoplasmic face (right). (**a**) Inward facing (I_f_, pdb 4ZP0^64^), (**b**) outward open (O_o_, pdb 6GV1^66^).

We used DEER to elucidate the solution conformation of MdfA solubilized in detergent, in light of the recently published crystal structures^64,66,68^. For this purpose, we have labeled a series of double cysteine MdfA mutants with both MTSSL and C2-Gd, a Gd(III) tag (Fig. 1)^69,70^. Our studies included 13 double mutants labeled with both MTSSL and C2-Gd; eight of them designed to probe the periplasmic face of MdfA, and five, the cytoplasmic region. In addition, one more C2-Gd(III) and one more MTSSL labeled constructs were prepared and characterized. This labeling approach is similar to that used on the pioneering DEER application to lactose permease, LacY^46^. Distance distributions were evaluated to assess agreement with the two published crystal structures (see Fig. 2). Both spin labels show that the periplasmic side of detergent-solubilized MdfA has a structure more similar to the O_o_ conformation, while for the cytoplasmic face, the DEER distances show only a subtle preference for the O_o_ structure. To the best of our knowledge, this is the first extensive comparison of the two types of labels and it reveals a relatively general agreement between distances determined from the two types.

## Results

### Construction and characterization of spin-labeled variants of MdfA

Guided by the crystal structure of MdfA(Q131R)^64^, we modified several sites at the cytoplasmic or periplasmic edges of the TMs by cysteines (Fig. 2A), in the background of an active cysteine-less version of MdfA^71^. Initially, we assessed the Mdr activity of single cysteine mutants (Fig. S1) and those that retained function were selected for further analyses, including expression tests (Fig. S2A) and MTSSL accessibility (Fig. S2B). Single cysteine mutations were combined to generate double cysteine mutants at sites that did not compromise function and enabled efficient labeling at distances amenable to DEER measurements. Nine double cysteine mutants on the periplasmic side and six on the cytoplasmic side were selected based on their level of expression (Figs 2 and S3A) and Mdr activity (Fig. S3B). These mutants were overexpressed, purified as described earlier^67^, and labeled with either MTSSL or the C2-Gd tag (Fig. 1). Labeled samples were flash-frozen in liquid N_2_ for EPR studies. Table S1 in the Supplementary Information (SI) lists all the double mutants and their identification in terms of transmembrane helix location.

### DEER measurements

**Figure 3:**
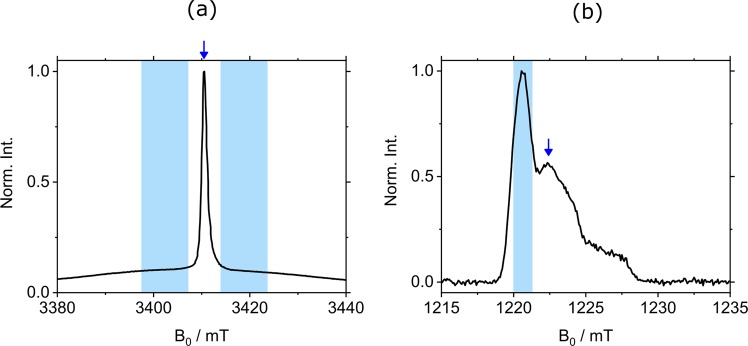
Echo-detected EPR spectrum of (**a**) MdfA TM5^163^–TM9^307^ labeled with C2-Gd (10 K, W-band). The positions and range of the chirp pump pulses are indicated in light blue and the position of the observe pulses are indicated by an arrow. (**b**) MdfA TM5^163^–TM9^307^ labeled with NO (83 K, Q-band). The region affected by pump pulse is indicated in light blue, and the observe position is indicated by an arrow.

In order to compare the various constructs and the different spin labels, all the EPR experiments were conducted at slightly alkaline pHs (7.2–7.5), under which the functional acidic residues are likely deprotonated^72^. Eight MdfA double cysteine variants were labeled in the periplasmic side with MTSSL, in the following referred to as NO, and C2-Gd, for a total of 16 constructs. Additionally, another double-mutant was labeled with only C2-Gd. Five pairs on the cytoplasmic side were labeled with both labels and one more with NO only (see Table S1). Examples of W-band and Q-band echo-detected EPR spectra of MdfA labeled with C2-Gd and NO, respectively, are shown in Fig. 3 with indicated positions of the pump and observe pulses in the DEER sequence. The C2-Gd labeled samples gave rather similar phase memory times in the range of 1.0–1.6 µs, allowing DEER evolution times as long as 4–5 μs.

**Figure 4:**
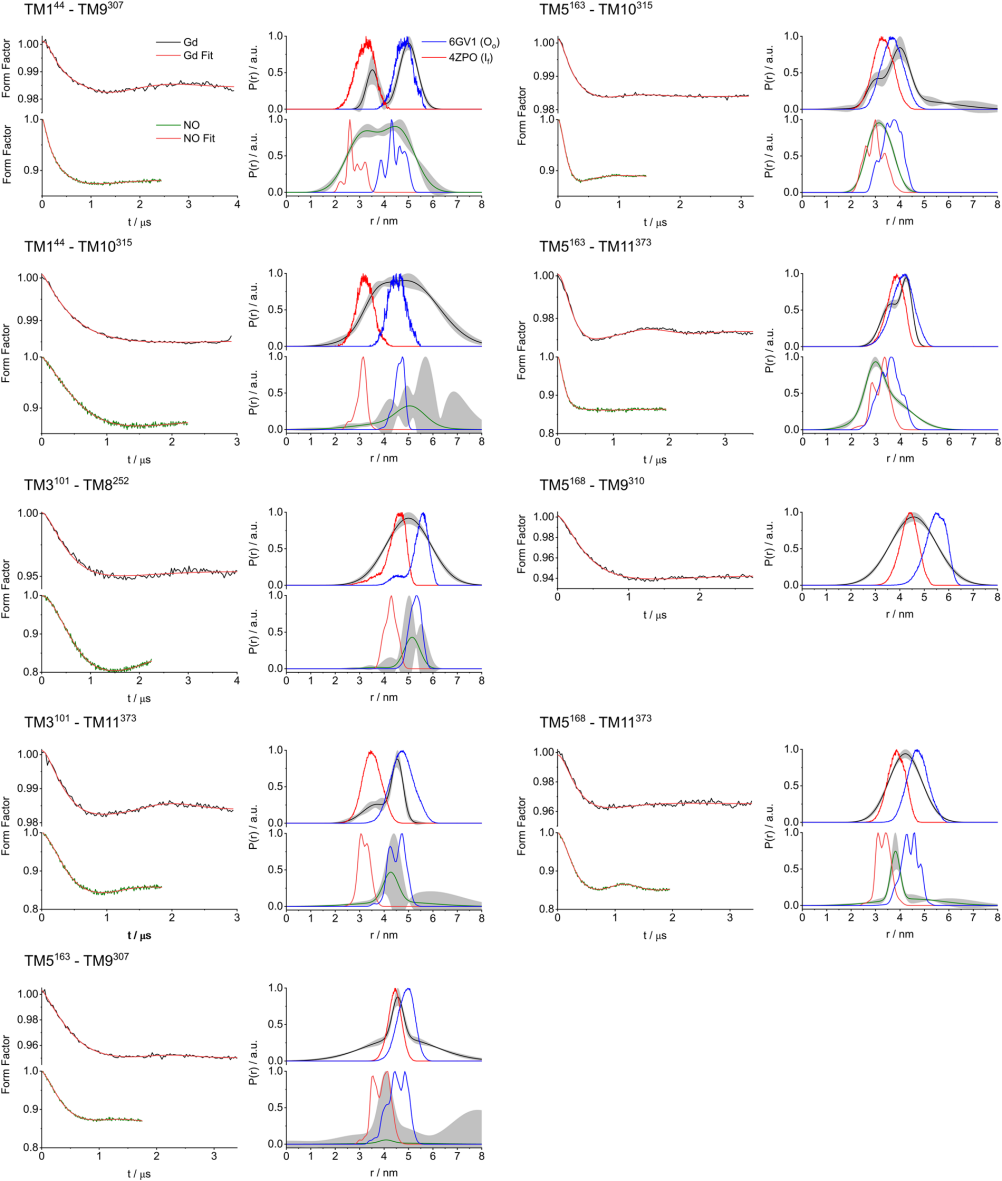
DEER data for the periplasmic spin label pairs. (left panels) DEER form factors for MdfA labeled with either C2-Gd (upper panels, black traces) or NO (lower panels, green traces). Red traces indicate the fit using the distance distribution on the right; (right panels) DEER-derived distance distributions. The results were analyzed using DD and compared to a distance distribution calculated from the crystal structures 4ZP0 (I_f_, red) and 6GV1 (O_o_, blue). The shaded gray area denotes the confidence bands for the distance distribution.

**Figure 5:**
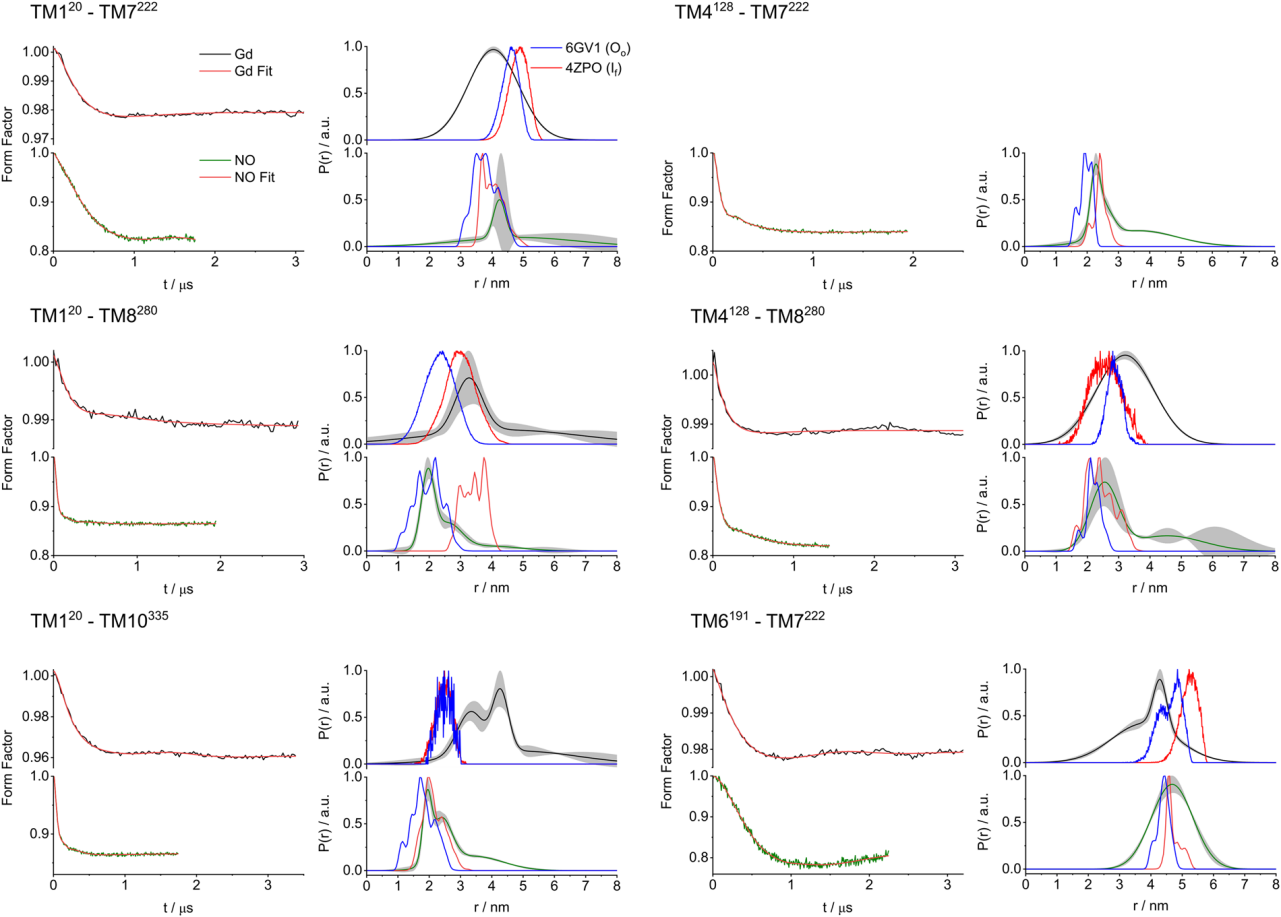
DEER data for the cytoplasmic spin label pairs. (left panels) DEER form factors for MdfA labeled with either C2-Gd (upper panels, black traces) or NO (lower panels, green traces). Red traces indicate the fit using the distance distribution on the right; (right panels) DEER-derived distance distributions. The results were analyzed using DD and compared to a distance distribution calculated from the crystal structures 4ZP0 (I_f_, red) and 6GV1 (O_o_, blue). The shaded gray area denotes the confidence bands for the distance distribution.

Figures 4 and 5 show the NO-NO and Gd(III)-Gd(III) DEER form factors obtained after background removal and the derived distance distributions of all measurements on the periplasmic and cytoplasmic side of the protein, respectively. The primary DEER data with the background correction function are shown in Figs S4 and S5. The data quality for the two types of labels is similar in terms of DEER signal-to-noise ratio (SNR), with the low modulation depth observed for C2-Gd compensated by the large echo intensity and enhanced by the use of an Arbitrary Waveform Generator (AWG) to produce shaped (linear chirp) pump pulses^37,73,74^. The distance distributions shown in Figs 4 and 5 were derived by model fitting to Gaussians using the DD software^75^. The results are similar to those derived by Tikhonov regularization obtained using the DeerAnalysis software^2^ (Figs S6 and S7). The DEER traces recorded on MdfA labeled with C2-Gd were typically longer (3.0–3.5 µs) than their NO counterparts (1.4–2.2 µs) owing to the longer distances of MdfA labeled with C2-Gd^76^. The data were analyzed to assess: (i) the agreement between the NO and C2-Gd results and (ii) their agreement with the distance distributions calculated using the crystal structures. In these comparisons we mostly referred to the maximum of the DEER distance distribution, represented by r_max_, and in cases where the distance distribution was bimodal, we considered the major component. Table S1 lists all the r_max_ values.

#### Comparison of MdfA-C2-Gd and MdfA-NO distance distributions

**Figure 6:**
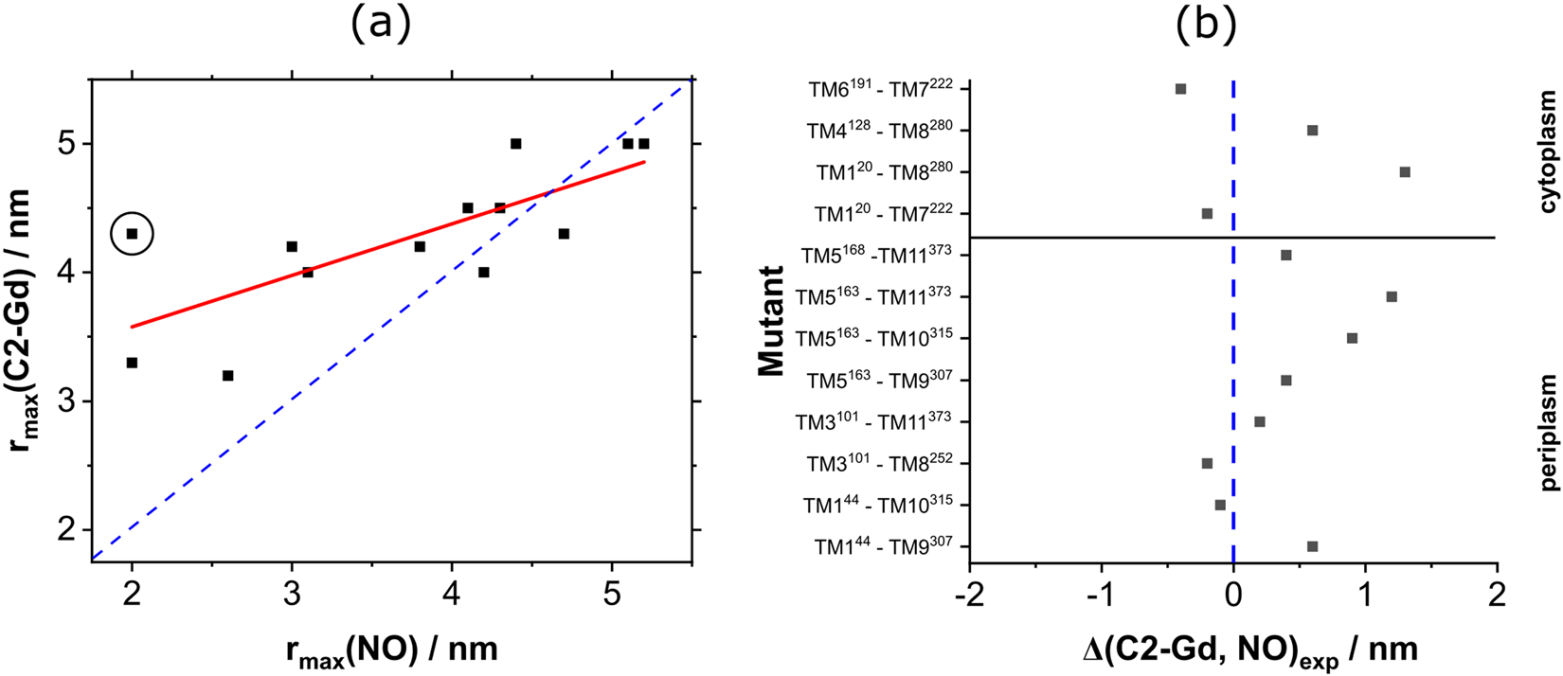
(**a**) Plot of r_max_ for the C2-Gd labeled mutants vs r_max_ for the NO labeled ones. When more than 1 Gaussian appeared in the distance distribution, we considered only the major one. The blue, dashed line corresponds to a perfect match, whereas the red line is a linear fit. Mutant TM1^20^–TM10^335^, which was removed from subsequent analysis as described in the text, is indicated by a black circle. (**b**) Plot of Δ(C2-Gd, NO)_exp_ for each mutant studied, except for TM1^20^–TM10^335^.

C2-Gd and MTSSL differ in a number of properties that affect the DEER traces and consequently the resulting distance distributions. First, the distance between the spin bearing moiety and the cysteine sulfur in C2-Gd is longer than in MTSSL (0.93 nm vs 0.69 nm for an extended label conformation). Second, C2-Gd is larger than the MTSSL, and is positively charged compared to the hydrophobic neutral MTSSL. Yet, despite these different properties, Figs 4 and 5 show that in most cases the distance distributions measured with the two labels report similar structural information. A general trend of broad distance distributions emerged for both labels consistent with the expected conformational heterogeneity of MdfA. Figure 6a shows a correlation plot where the r_max_ values for MdfA labeled with C2-Gd are plotted against those of MdfA labeled with NO (considering r_max_ of the major distance component). While the correlation improves for longer distances, we observed notable differences for short distances. These differences could partially arise from the effect of the pseudo-secular terms of the dipolar interaction for short distances and small zero-field splitting although these were shown to mostly broaden the distance distribution rather than shifting the average distance^20,77^. Moreover, local perturbations due to the size of the Gd(III) label are expected to be independent of the distance between the labels.

Figure 6b shows the difference between the position of the main distance distribution peak of NO and that of C2-Gd, Δ(C2-Gd,NO)_exp_ = r_max_(C2-Gd)-r_max_(NO). This plot highlights that, in most cases, r_max_ for C2-Gd is larger as expected, but there is a variation in Δ(C2-Gd,NO)_exp_, on the range of 0–1.2 nm with large deviation for the construct TM1^20^–TM10^335^. For four constructs we observed C2-Gd distances slightly shorter than those of the NO counterparts with TM6^191^-TM7^222^ showing the largest difference of 0.4 nm, indicating significant contributions from the labels’ relative orientations, conformation and local repacking. Correlation plots derived from distance distributions obtained by Tikhonov regularization show the same trend (Fig. S8). We observed that the construct TM1^20^–TM10^335^, which showed a large difference between C2-Gd and NO, also exhibits a large deviation between the calculated and experimental distance distribution for C2-Gd, while for the NO results match reasonably well (see below). We suspect that the steric accommodation of the large Gd-C2 label at the buried TM10^335^ position is at the origin of this deviation. Thus, data from this mutant with C2-Gd will not be included further in the analysis.

Comparison of the width of the distance distributions for the two labels did not reveal a discernable overall trend suggesting that the width may be context dependent. For example, the distance distribution reported by the NO for TM1^44^–TM9^307^ is significantly broader than that of C2-Gd. In contrast for TM5^168^–TM11^373^ and TM3^101^–TM8^252^ the trend is the opposite, with C2-Gd showing a broader width, while the width is comparable for the other pairs. We note that for distance distributions below 4 nm there could be artificial broadening contributions to the distance distributions of C2-Gd due to deviations from the weak-coupling approximation^20,78^.

#### Comparison of distance distributions with the crystal structures

To determine whether the structure of MdfA solubilized in detergent micelles in the apo state at pH 7.2–7.5 resembles either of the two crystal, we compared the experimental distance distributions with calculated distance distributions from the two crystal structures 4ZP0 and 6GV1 which are referred to as I_f_ and O_o_, respectively (see Figs 4 and 5). The predicted distance distributions for the NO pairs were obtained using MMM^79^, whereas those for C2-Gd pairs were obtained using a different approach described in the experimental section. Correlation plots of the predicted r_max_(NO) vs. predicted r_max_(C2-Gd) for each of the crystal structures of MdfA, I_f_ and O_o_, (Fig. S9) show that the calculated C2-Gd distances are larger than the corresponding NO distances, with a spread of 0 ≤ Δ(Gd,NO) ≤ 1 nm.

**Figure 7:**
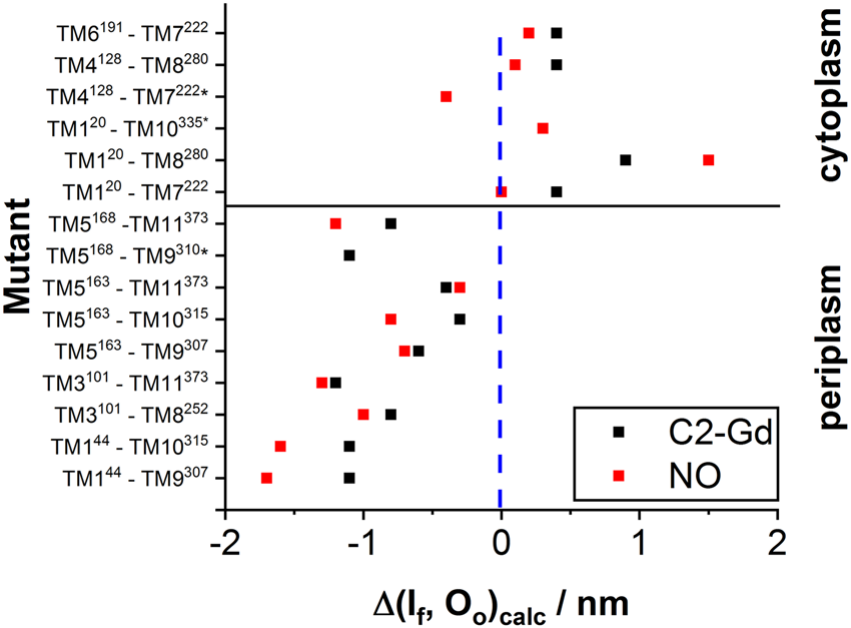
Plots of Δ(I_f_, O_o_)_calc_ for each of the mutants studied. It shows the expected change in the distance distribution between the two crystal structures. Pairs for which Δ(I_f_, O_o_)_calc_~0 are not sensitive in distinguishing between the two crystal structures. For mutants indicated with an asterisk (*), only NO or C2-Gd experimental data are available.

To highlight the differences between the two structures expected for each spin label pair, plots of Δ(I_f_, O_o_)_calc, Gd_ = r_max_(I_f_, C2-Gd)_calc_ − r_max_(O_o_, C2-Gd)_calc_ and Δ(I_f_, O_o_)_calc, NO_ = r_max_(I_f_, NO)_calc_ − r_max_(O_o_, NO)_calc_ are shown in Fig. 7. In the periplasm, there is a general trend towards larger distances in the O_o_ structure that is reflected by segregated negative data points. Differences above 1 nm are expected for 4 mutants labeled with NO and 3 mutants labeled with C2-Gd. In general, the calculated NO distances span a larger difference range than the C2-Gd distances, suggesting that it is more sensitive to the structural difference between I_f_ and O_o_. In contrast, the differences are less conspicuous for the cytoplasmic mutants, where only one mutant, TM1^20^–TM8^280^, is expected to exhibit a large difference. Here however, Gd-C2 is more sensitive to the change, except for TM1^20^-TM8^280^. Thus, opening/closing of MdfA is less pronounced in the cytoplasmic region, at least for the selected pairs. Figure 7 highlights the advantages of using two different spin labels, showing that for a particular pair, the two spin labels can show different sensitivities to differences between the structures, such as in the case of TM1^20^-TM8^280^_._

**Figure 8:**
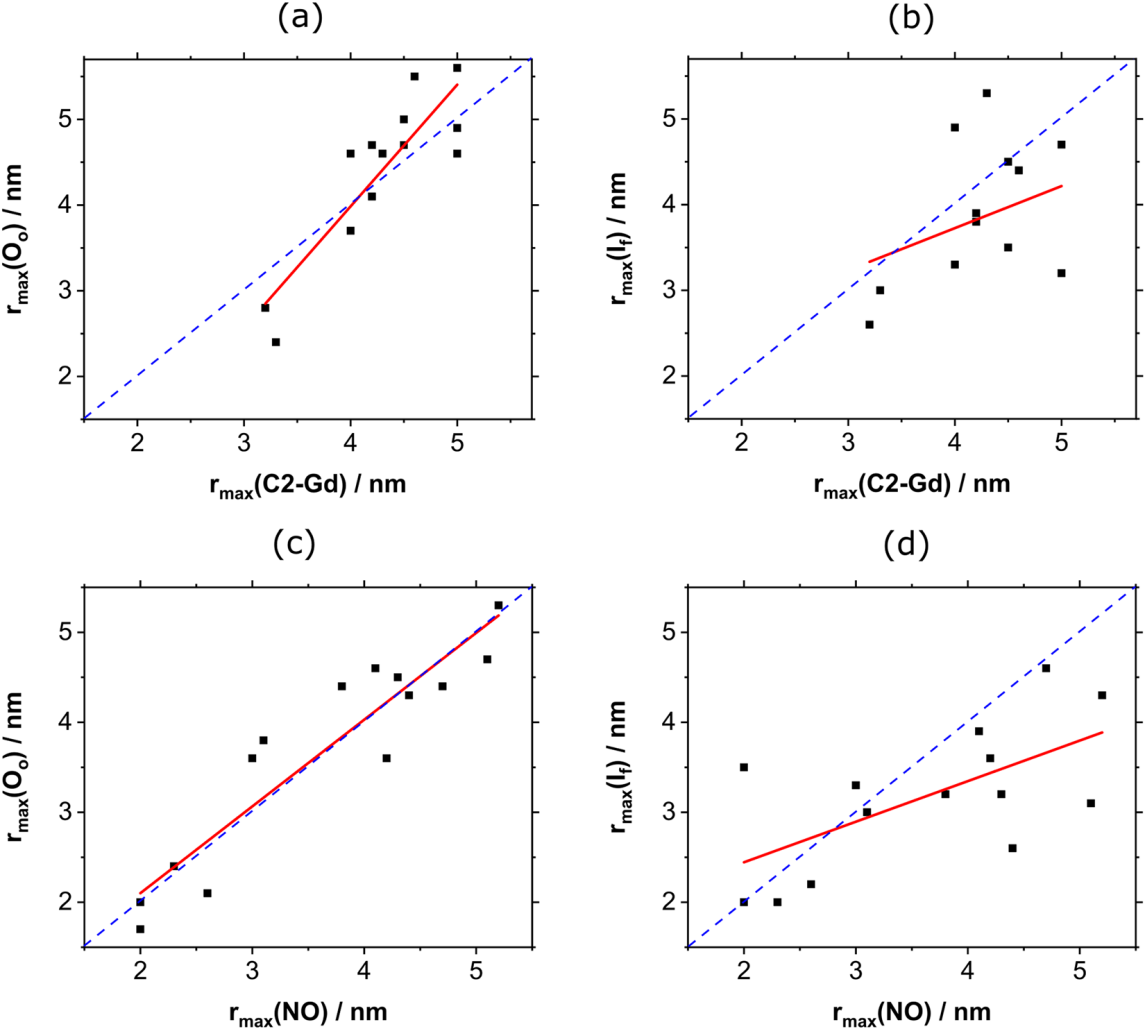
Comparison of the experimental and crystal structure derived r_max_ values for C2-Gd and r_max_ calculated from the (**a**) O_o_ structure, (**b**) I_f_ structure and the same for the NO labels (**c,d**) respectively. When more than one Gaussian appeared in the distance distribution, we considered only the major one. The dashed blue line corresponds to a perfect match, whereas the red line is a linear fit.

In order to assess the correlation between the experimental data and the crystal structures, we plotted the calculated r_max_ from the crystal structures against the r_max_ from the measured data (see Fig. 8). We observed a more robust agreement with the O_o_ crystal structure for both spin labels (panels a and c). For NO a linear fit gave a slope of 0.96 and a R^2^ of 0.86, which is close to the slope of 1, as would be expected for direct correspondence. For C2-Gd, the correlation is less ideal, yielding a slope of 1.42 with a R^2^ of 0.76. The correlations with the I_f_ structure, shown in panels b and d, yielded a slope of 0.49 with an R^2^ of 0.11 for C2-Gd and a slope of 0.45 with an R^2^ of 0.40 for NO, indicating a quantitative agreement between the DEER data and the O_o_ conformation, but not the I_f_ conformation.

**Figure 9:**
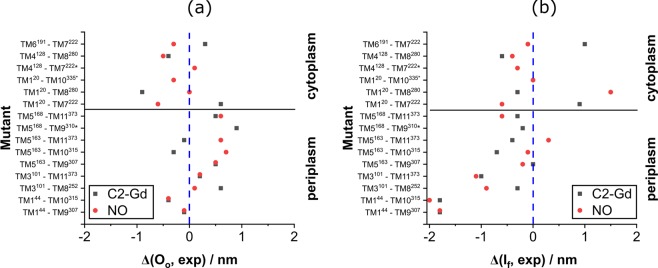
Plots showing the difference between the predicted and experimental r_max_ values (**a**) for the O_o_ structure for the mutants studied (red symbols are for C2-Gd, Δ(O_o_, exp)_C2-Gd_, and black for NO, Δ(O_o_, exp)_NO_); (**b**) the same for the I_f_ structure (Δ(I_f_, exp)_C2-Gd_, (Δ(I_f_, exp)_NO_). The vertical dotted blue lines mark the zero lines (perfect agreement) and the horizontal black lines represent the division between periplasmic and cytoplasmic pairs. The mutants marked with * were labeled with only one type of spin label.

To allow for a more detailed comparison with the crystal structures, Fig. 9 highlights the differences between the crystal structures and the experimental r_max_ values for each spin label pair, Δ(O_o_, exp)_C2-Gd_ = r_max_(O_o_, C2-Gd)_calc_ − r_max_(C2-Gd)_exp_, Δ(O_o_, exp,)_NO_ = r_max_(O_o_, NO)_calc_ − r_max_(NO)_exp_ and accordingly for I_f_. Several general trends are noted: (i) the overall deviation from the crystal structure (indicated by larger absolute values) is larger for the periplasmic face of the I_f_ structure, and the scatter between NO and C2-Gd data is comparable. (ii) The correspondence with the structures is more similar for the cytoplasmic face mutants.

Comparison to the O_o_ crystal structure (Fig. 9a) shows that for the periplasmic pairs the majority of the points are positive, indicating a more closed conformation of detergent-solubilized MdfA compared to the crystal structure. The C2-Gd labeled TM5^168^–TM9^310^ is identified as the largest outlier on the periplasmic side (0.9 nm). Also notable are the constructs TM5^163^–TM10^315^ and TM1^20^–TM7^222^, on the periplasmic and cytoplasmic sides, respectively, which exhibit the largest difference between NO and C2-Gd distances. On the cytoplasmic face, the majority of the points are negative, suggesting a more open conformation as compared to the O_o_ crystal structure. Here the C2-Gd labeled mutant TM1^20^–TM8^280^ (~0.9 nm) as well as the NO-labeled TM1^20^–TM7^222^ (~0.6 nm) display the largest deviation.

Figure 9b shows the same data for the I_f_ crystal structure. In contrast to O_o_, most points are negative indicating longer distances in the periplasmic side for the detergent solubilized conformations. The largest absolute deviation in the periplasm was found for mutants TM1^44^–TM9^307^ and TM1^44^–TM10^315^ (~1.8 nm each) for C2-Gd and, TM1^44^–TM10^315^ (~2.0 nm) for NO. The largest scatter between NO and C2-Gd in the periplasm is observed for the mutant TM5^163^–TM11^373^ (0.7 nm) and is in the same order of magnitude as for the O_o_ conformation. In the cytoplasm, most of the points are negative, with three on the positive side with large deviations. TM1^20^–TM8^280^ yields the largest absolute deviation (1.5 nm) as well as the largest Δ(C2-Gd, NO)_exp_ scatter (1.8 nm). TM1^20^–TM7^222^, which showed the largest scatter in the O_o_ conformation, shows a similarly large deviation at 1.5 nm. While a clear distinction between the two structures was observed for the periplasmic pairs, the cytoplasmic pairs show only a slight preference towards the O_o_ structure, suggesting a somewhat more open conformation. This is in agreement with Fig. 7, which shows that the difference between the structures for the cytoplasmic pairs is expected to be small.

Interestingly, while for TM5^163^–TM9^307^ both labels deviate significantly from the O_o_ predictions (0.5 nm for both C2-Gd and NO), they agree with the I_f_ prediction (Fig. 9b). The spin label pairs that are predicted to show the largest differences between the structures are TM1^44^–TM9^307^, TM1^20^–TM8^280^ and TM3^101^–TM11^373^ (see also Fig. 7). Out of these only TM3^101^–TM11^373^ gives an excellent agreement with O_o_ and a poor one with I_f_ for both labels. Similar discrimination is reported by NO-labeled TM1^20^–TM8^280^, but not its C2-Gd counterpart. We also compared the experimentally determined distance distributions calculated with Tikhonov regularization (Figs S10 and S11), and obtained similar results, reinforcing our conclusion that that the DEER measurements agree significantly better with the O_o_ structure, as compared to the I_f_ structure for the periplasmic face.

## Discussion

In this work, we have explored the conformation of MdfA solubilized in detergent at pH = 7.2-7.5. We used two different spin labels with very different chemical properties and sizes in order to eliminate label-specific effects on the distance distribution and to test the applicability of Gd(III) spin labeling to membrane proteins in a systematic way. The results reported by the two labels are consistent and show that in the apo state MdfA favors the O_o_ crystal structure^66^. Nonetheless, there are some notable differences between the predicted and the experimental distance distributions: (i) While the periplasmic pairs show a clear preference for the O_o_ structure, the cytoplasmic pairs exhibit only a marginal preference for the O_o_ structure (Fig. 9); (ii) The periplasmic region seems to be more “closed” in detergent solution than in the O_o_ crystal structure, whereas the cytoplasmic side is more open. This opposite movement of the periplasmic and cytoplasmic faces is consistent with the alternating access model found earlier on for LacY^46^ and it shows that in the apo state the protein solubilized in detergent is in a conformation ready for the uptake of the substrate. (iii) The largest deviations for both labels were observed with TM5^163^–TM9^307^ and TM5^168^–TM11^373^, and for TM5^168^–TM9^310^ which was labeled only with C2-Gd. This is not surprising, because residues 163 and 168 are located at the periplasmic edge of TM5, which was proposed to exhibit major movements during the transition from the I_f_ conformation to the O_o_ one^66^. On the cytoplasmic face, distance distributions for the TM1^20^–TM7^222^ pair exhibit the largest discrepancy between C2-Gd and NO and are incongruent with the predicted distance distributions of either O_o_ or I_f_ crystal structures, indicating the possibility of spin label distortion of local structure. We suspect that the large size of the C2-Gd label led to a significant structural distortion at the cytosolic site TM10^335^, which is located close to the putative recognition pocket.

The differences between the crystal structures and the measured DEER distances in detergent-solubilized MdfA may reflect the imposed constraints on the protein in the crystalized state. In the O_o_ structure this could be the Fab fragment, which freezes the cytoplasmic face in a certain conformation. In the I_f_ crystal structure the insertion of a rather inactivating mutation^67^ possibly locked the protein in an inactive crystallisable conformation. In both cases, the crystal structures might not represent the most probable natively occurring steady state conformations of MdfA.

While the discussion above focused on r_max_ values, the width of the distance distribution is another parameter that can report directly on flexible, disordered parts of the transporter. Large deviations from the calculated width, which account for the contributions of the flexibility of the spin-label tether, may indicate local disorder in the protein. The calculated distance distribution (Figs 4 and 5) shows that for C2-Gd the width is somewhat larger (gross estimates, 20–50%) than for NO except for TM1^44^–TM9^307^. In general, the experimental distance distributions are broader than the calculated ones, particularly for the cytoplasmic pairs (except for TM1^20^–TM8^280^), suggesting increased flexibility on this side of the transporter. There are cases where the two spin labels exhibit significant width, and others such as TM5^168^–TM11^373^ and TM3^101^–TM8^252^ that are broader for C2-Gd and TM1^44^–TM9^307^ and TM5^163^–TM11^373^ that are broader for NO. TM3^101^–TM11^373^ displays a relatively narrow distance distribution, in agreement with the calculated one. Finally, lipids have been demonstrated to shift the equilibrium between conformational states^54^, thus, a detailed investigation of MdfA conformational dynamics will require studies in membrane mimetics such as liposomes or nanodiscs.

## Conclusions

DEER measurements on a series of double mutants of MdfA labeled with two different spin labels, nitroxide and C2-Gd, were carried out in order to compare the conformation and flexibility of MdfA solubilized in detergent solution to the two crystal structures. To our knowledge, this study represents the most extensive comparison of DEER data, obtained with Gd(III) and nitroxide spin labels, on a protein. Both labels, which are dissimilar in their structures, sizes, charge and hydrophobicities, yielded consistent results. In addition to revealing the flexibility of detergent-solubilized MdfA, the results show that the periplasmic side has a similar structure to the O_o_ conformation reported by the 6GV1 crystal structure, though somewhat more closed, and is quite different from the I_f_ crystal structure. For the cytoplasmic face the results show only a slight preference for the O_o_ with a more open conformation.

## Methods

### Strains, plasmids, and mutants

All experiments were conducted with *E*. *coli* UTL2^80^ derivative (UTL2*mdfA::Kan*). Plasmid pT7-5/200us/*CL-mdfA-6His* was used for resistance, expression and analysis of protein labeling experiments. Plasmid pUC18/Para/*CL-mdfA-6His* was used for overexpression. All mutants were generated using a standard PCR method with mutagenic oligonucleotide primers and a plasmid encoding cysteine-less (CL) MdfA as a template^71^. The plasmids were verified by sequencing.

### Drug resistance assay

Antibacterial resistance was assayed as described^81^. Briefly, *E*. *coli* UTL2*mdfA::Kan* harboring empty vector or expressing MdfA variants were grown aerobically to *A*_600_ of ~1 and a series of 10-fold dilutions was prepared. 4 μl of the serial dilutions were spotted on drug-containing LB-agar plates and tested for growth after overnight incubation at 37 °C.

### Expression analysis

Overnight cultures of *E*. *coli* UTL2*mdfA*::*Kan* harboring empty vector or expressing CL-MdfA or variants were diluted 1:100 and grown to A_600_ of 0.8–1. Cells were harvested and resuspended in buffer A (20 mM Tris-HCl, 500 mM NaCl, 10% glycerol), supplemented with 1 mM PMSF. Cells were sonicated three times at 50%, 10 s on, 10 s off and debris were removed by centrifugation (5 min, top speed in a table-top centrifuge, at 4 °C). The supernatant was then centrifuged at 244,000 g for 30 min to pellet the membranes. Membranes were resuspended in buffer A and the total membrane protein concentration was analyzed by Lowry. Samples (20 µg or total membrane protein) were then subjected to 12.5% (w/v) SDS/PAGE, electroblotting, and detection using India HisProbe-horseradish peroxidase (Pierce) and ECL.

### MdfA expression and purification

For all experiments, *E*. *coli* cells, harboring plasmid pUC18/*Para*/CL-*mdfA-6His* or any of the mutated constructs, were grown at 37 °C in LB medium supplemented with ampicillin (200 μg/mL) and kanamycin (30 µg/ml). Overnight cultures were diluted to ~ 0.05 *A*_600_ units, grown to 0.8 *A*_600_ units, and induced with 0.2% (w/v) arabinose for 1.5 h at 37 °C. For C2-Gd experiments, *E*. *coli* cells were grown at 37 °C in LB supplemented with ampicillin (200 μg/mL) and kanamycin (30 μg/mL) until an OD of 0.8 was reached, transferred to 16 °C and induced with 0.2% (w/v) arabinose overnight. Exceptions were made for mutants TM5^163^–TM10^315^, TM1^20^–TM7^222^, and TM1^20^–TM10^335^ as part of an upgraded protocol. Here, *E*. *coli* cells were grown at 37 °C in Terrific Broth rich medium supplemented with ampicillin (200 μg/mL) and kanamycin (30 μg/mL) overnight. Subsequent induction was performed with 0.2% (w/v) arabinose for 1.5 h at 37 °C. Cell pellets were centrifuged for 30 min at 6,000 g and resuspended in 50 mM KPi pH 7.3 supplemented with 2 mM MgSO_4_. DNAse, 10 μg/mL, and PMSF, 1 mM, were then added and the cells passed three times through a pressure cell homogenizer (Stansted) at 15 kPsi for disruption. Cell debris was removed by centrifugation (15 min, 20,000 g), and the membranes were collected by ultracentrifugation (1 h, 167,000 g). The membranes were suspended by homogenization in 20 mM tris-HCl pH 8.0, 0.5 M NaCl, 10% glycerol, then snap-frozen in liquid nitrogen and stored at −80 °C.

For MdfA purification, 5 mM of imidazole and 2 mM of β-mercaptoethanol were added to thawed membranes, which were then solubilized by addition of 20% n-dodecyl-β-D-maltopyranoside (DDM, Anatrace) to a final concentration of 1.1%. Insoluble material was discarded by ultracentrifugation (30 min, 167,000 g) and the soluble fraction was mixed with solubilization buffer-equilibrated *Talon* beads (Clontech) (typically 0.5 mL bed volume for 2.5 gm of thawed membranes). Next, the mixture was agitated for 2 hr at 4 °C and the suspension was poured into a column and washed with 30 column volumes of solubilization buffer (20 mM tris-HCl pH 8.0, 0.5 M NaCl, 10% glycerol, 5 mM imidazole, 0.1% DDM). MdfA was eluted in 20 mM tris-HCl pH 7.2 or 7.5, 0.12 M NaCl, 10% glycerol, 0.1% DDM, 200 mM imidazole. Protein concentration was determined by measuring *A*_280_ (1 mg/mL ~ 2.1 *A*_280_)^82^.

### Labeling with MTSSL and preparation of DEER samples

Labeling was done by adding 20-fold molar excess of MTSSL (100 mM, in DMF) to the eluted protein. The sample was then incubated at room temperature (RT) for 1.5 h after which 20-fold molar excess of MTSSL was added again, and the protein was incubated for an additional 1 h at RT. The sample was kept on ice overnight. The protein was loaded onto Superdex 200 10/30 GL (GE Healthcare) size exclusion chromatography column in 20 mM tris-HCl pH 7.5, 120 mM NaCl, 10% glycerol, 0.03% DDM. The protein was then concentrated to 75–100 μM using a 100 K MWCO concentrator (Amicon) and glycerol was added to a final concentration of 23.78% (v/v). SEC elution profiles are shown in Fig. S12. In each case, the major monodispersed fraction was collected and used for the experiment.

### Labeling with C2-Gd and preparation of DEER samples

C2 was synthesized as reported earlier^69,70^. Labeling was done by adding 10-fold molar excess of the C2 label previously loaded with Gd^3+^ (50 mM in H_2_O) to the eluted protein. The sample was incubated for 12 h at 4 °C followed by 12 h of dialysis against dialysis buffer (20 mM tris-HCl pH 7.2, 0.12 M NaCl, 10% (v/v) glycerol, 0.01% DDM). In the next step the protein was incubated with *Talon* beads (Clonetech). Bed volume was in the range of 100–150 μl, depending on the volume of the sample after dialysis. The mixture was agitated for 2 h at 4 °C, washed, and consequently eluted with elution buffer (20 mM tris-HCl pH 7.2 or 7.5, 0.12 M NaCl, 10% glycerol-d8, 0.1% DDM, 200 mM imidazole, D_2_O). Protein concentration was determined by measuring *A*_280_ (1 mg/mL ~ 2.1 *A*_280_)^82^ and the protein concentrated to a final concentration of 20–50 μM using a 100 K MWCO concentrator (Amicon) and the sample loaded in 0.6 mm quartz capillaries.

### Analysis of protein labeling

Membranes were prepared as described above, the total membrane concentration was adjusted to 3.5 µg/µl and aliquoted to 400 µl. Membranes were then solubilized by adding 20% DDM to a final concentration of 1.1% and incubated for 2 h at 4 °C with tilting. Solubilized protein was recovered by centrifugation (100,000 g, 30 min, 4 °C), and divided into two equal portions. MTSSL (50 mM, 50% DMSO) was added to one of the portions at a final concentration of 1.25 mM and the same volume of 50% DMSO without MTSSL was added to the second portion. The two samples were then incubated at RT for 2 hr, after which 20 µl of suspended *Talon* beads (Clontech) were added to each, and incubated at 4 °C, for 1 h. Unbound protein was removed by centrifugation (700 g, 2 min) and the beads were washed twice with 300 µl of solubilization buffer. The protein was eluted with 2 × 32.5 µl of elution buffer (20 mM Tris-HCl pH 7.2, 0.12 M NaCl, 10% glycerol, 150 mM imidazole, 0.1% DDM). SDS was then added to both samples, at a final concentration of 1%, and Mal-PEG(5000) (Sigma) was added to a final concentration of 2 mM. Samples were incubated for 15 min at 37 °C, and for an additional 1 h and 45 min at RT in the dark. Non-reducing sample buffer was then added, incubated for 30 min at 37 °C, and 20 µl of the entire reaction was then subjected to 12.5% (w/v) SDS/PAGE, electroblotting, and detection using India HisProbe-horseradish peroxidase and ECL.

### DEER measurements

C2-Gd DEER experiments were performed on a home-built 95 GHz (W-band) EPR spectrometer equipped with two microwave channels and a 2 W solid-state amplifier^83,84^ using the controller software SpecMan^85^. Shaped pulses were generated by a *Chase Scientific DA12000* Arbitrary Waveform Generator (AWG) with a sampling rate of 2 GS/s in an incoherent manner. Details on the AWG set-up and performance have been published elsewhere^37,73^.

The C2-Gd DEER experiments were recorded at 10 K with (i) the standard dead-time free DEER sequence^86^ (ii) a 4-pulse DEER sequence where the pump pulse was replaced with a linear chirp^73,74^, referred to as AWG DEER or (iii) rDEER, where the shaped pump pulse is swept between the primary echo and the first refocusing pulse^37^. An 8-step phase cycling on the observer pulses was used; the observer pulse lengths were t_π, obs_ = 30 ns while the pump pulse lengths as well as other parameters varied. The experimental details on the different experiments are shown in Table S2.

NO DEER experiments were performed on E-580 Q-band EPR spectrometer (Bruker) at 83 K with the standard 4-pulse, dead-time free DEER sequence^86^. Two-step phase cycling on the observer pulses was used with observer pulse lengths of t_π/2,obs_ = 12 ns and t_π,obs_ = 24 ns.

The raw data was analyzed with DeerAnalysis 2018^2^ using Tikhonov regularization (Figures in the SI) and with the DD program (Figures in the main text)^3,75^. Validation was performed with DeerAnalysis 2018 using 10 trials of level 1.5 white noise, and 11 trials of background starting from 500 ns–700 ns up to 1400 ns–1600 ns (exact numbers depend on the trace length).

### Calculation of Gd-Gd distance distributions from crystal structures

To directly relate the measured DEER distance distributions to the crystal structures of MdfA, we modelled the most likely distance distributions between spin labels by taking into account the physicochemical properties of the tags using a procedure described previously^87^. First, the C2-Gd tag was grafted onto a single cysteine residue. Second, tag conformations were generated by random variation of the dihedral angles between the Cα atom and the amide of the of the ethylene thio linker of the C2-Gd tag, allowing free rotation around the N-C bond while restricting the sampling to staggered conformations around C-C bonds and dihedral angles of 90° or −90° for the S-S bond with an uncertainty range of ±10°. The protein coordinates were kept fixed and conformers with steric clashes between tag and protein were eliminated. To account for possible sidechain mobility of neighbouring residues in the structure, van der Waals radii in these calculations were scaled down by 10%. For each tag position 5000 random variations were calculated to obtain >400 conformers without steric clashes. Gd-Gd distance distribution were then calculated from all pair-wise distances of the Gd positions in the different tag conformations at two labelling positions.

## Supplementary information

Supplementary information

Supplementary information with highlights
