# Supplementary material for: Probing the solution structure of the E. coli multidrug transporter MdfA using DEER distance measurements with nitroxide and Gd(III) spin labels
Source: Sci Rep. 2019 Aug 29;9:12528. doi: 10.1038/s41598-019-48694-0 (PMC6715713; doi:10.1038/s41598-019-48694-0)
Supplement: Supplementary file 1 — Supplementary information [file 41598_2019_48694_MOESM1_ESM.pdf]

Supporting information

Probing the solution structure of the E. coli multidrug transporter MdfA using DEER distance measurements with nitroxide and Gd(III) spin labels

Eliane H. Yardeni[1#], Thorsten Bahrenberg[2#], Richard A Stein[3], Smriti Mishra[3]

Elia Zomot[1], Bim Graham[4], Kellie L. Tuck[5], Thomas Huber[6], Eitan Bibi[1*$], Hassane S. Mchaourab[3*$], Daniella Goldfarb[2*$]

[1]Department of Biomolecular Sciences, Weizmann Institute of Science Rehovot 76100, Israel.

[2]Department of Chemical Physics, Weizmann Institute of Science, Rehovot 76100, Israel.

[3]Department of Molecular Physiology and Biophysics, Vanderbilt University, Nashville, TN, USA.

[4]Monash Institute of Pharmaceutical Sciences, Monash University, Parkville VIC 3052, Australia

[5]School of Chemistry, Monash University, Wellington Road, Clayton, Victoria, Australia

[6]Research School of Chemistry, The Australian National University, Canberra, ACT 2601, Australia

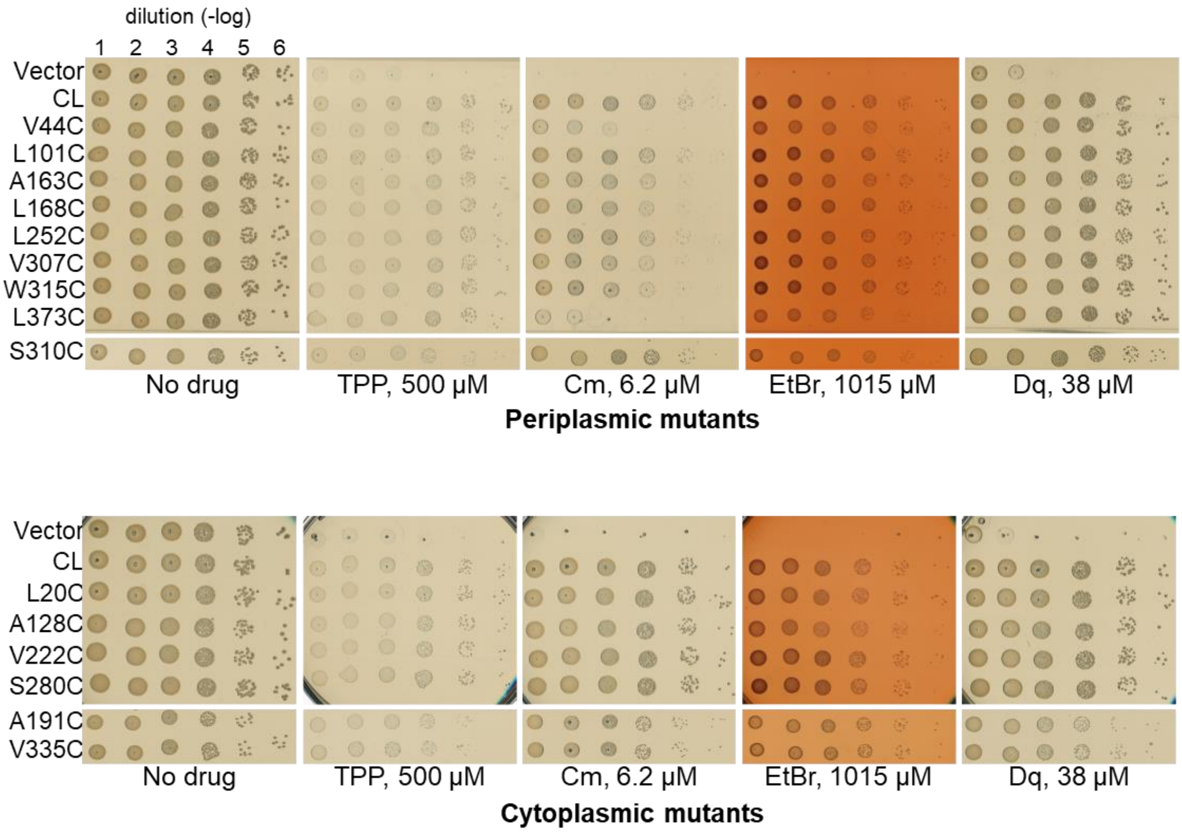

Figure S1: Mdr activity of single cysteine mutants. Serial dilutions of cells, expressing the indicated constructs (CL = cysteine-less) were spotted on LB-agar plates containing the indicated drugs. Upper panel, mutant S310C was analyzed in a separate plate, as shown by the space line. Lower panel, mutants A191C and V335C were analyzed in a separate plate, as shown by the space line.

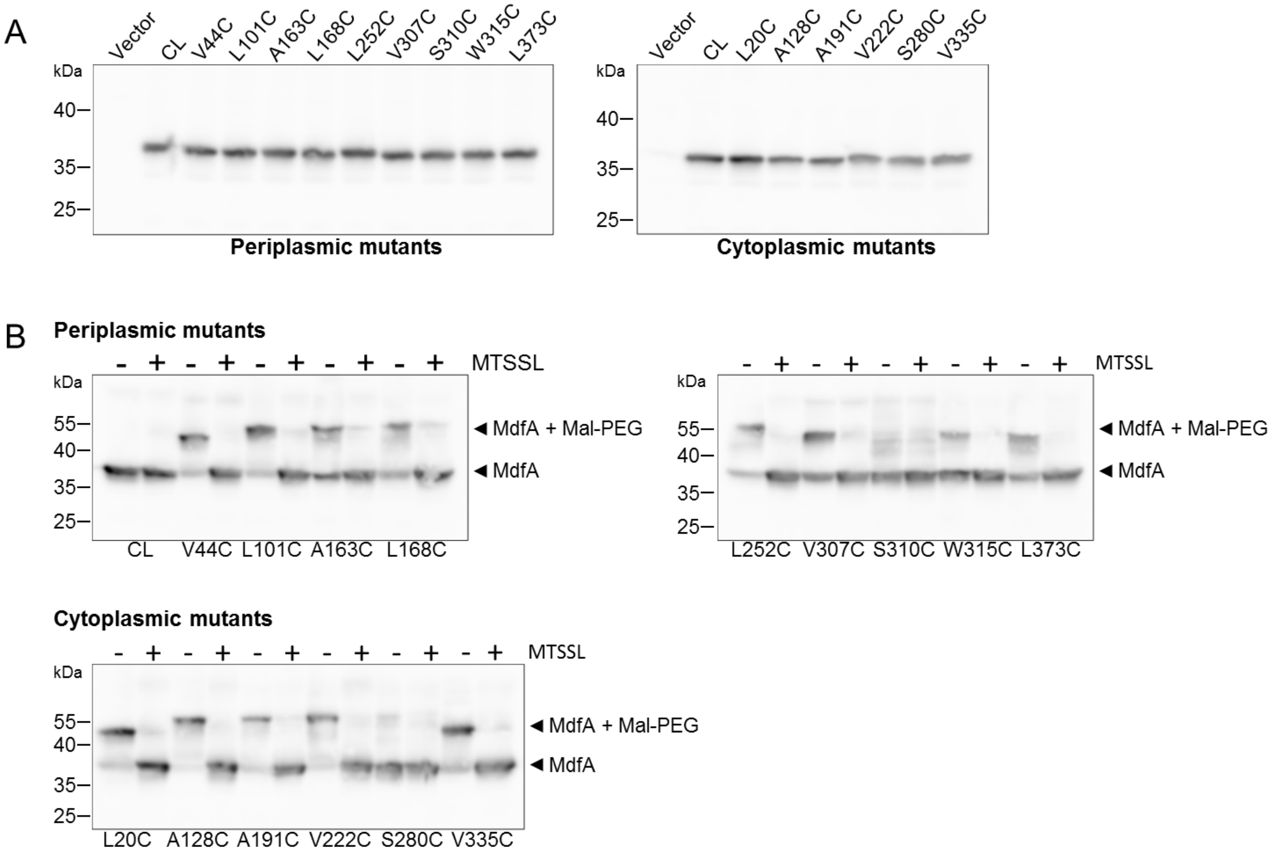

Figure S2: Expression and MTSSL-accessibility of the single cysteine mutants. **A.** Western blotting of membranes, prepared from cells expressing the indicated MdfA constructs. **B.** Cysteine accessibility to MTSSL. Solubilized, single cysteine mutants were incubated with (+) or without (-) MTSSL. After removal of non-bound label the samples were denatured with 1% SDS and incubated with Mal-PEG(5000).

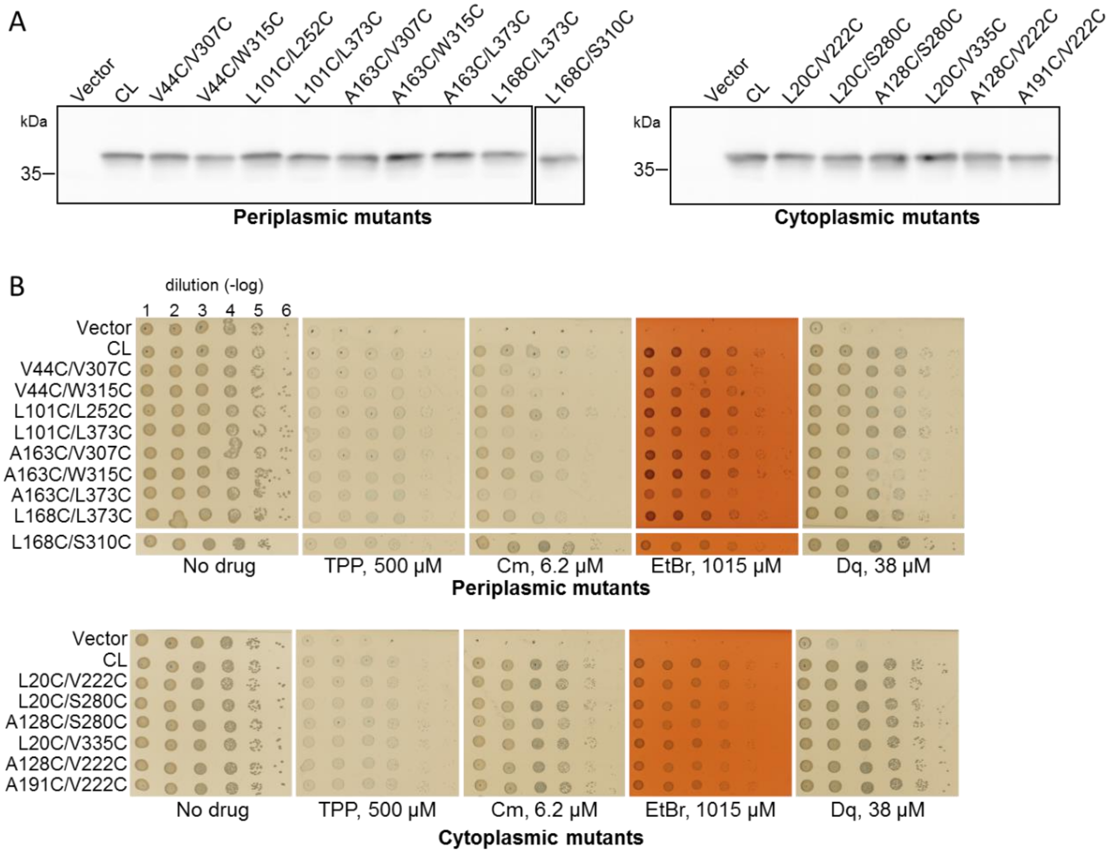

Figure S3: Characterization of double-cysteine mutants. **A.** Western blotting of membranes, prepared from cells expressing the indicated constructs. The expression of mutant L168C/S310C was analyzed in a separate gel, as shown by the separated frame. **B.** Cells expressing the indicated constructs were spotted on LB-agar plates containing the indicated drugs. Upper panel, mutant L168C/S310C was analyzed in a separate plate, as shown by the space line.

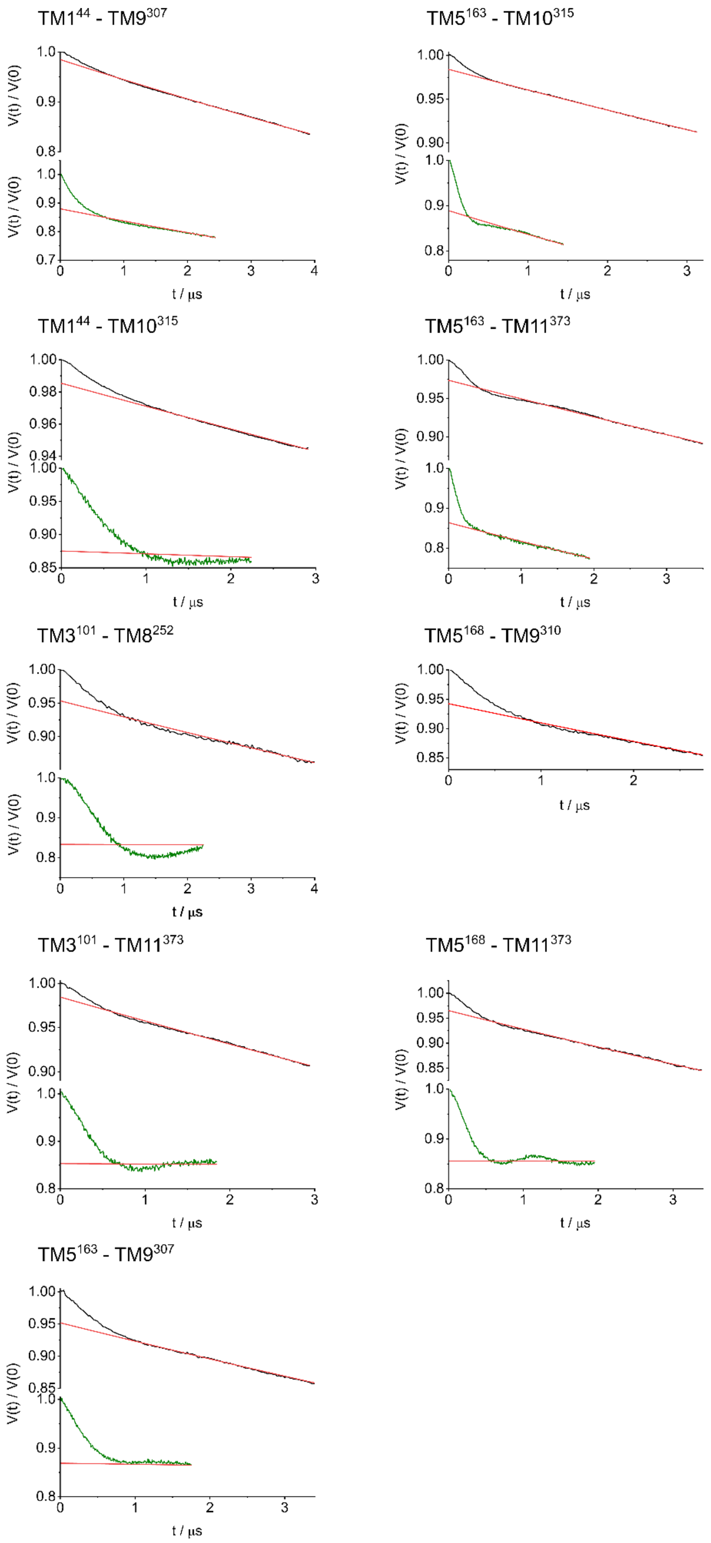

Figure S4: Raw data of all DEER experiments of periplasmic constructs shown in Fig. 4. For each mutant, the top panel shows Gd(III) data (black trace) and the bottom panel MTSSL data (green trace). The background decay obtained by the DD software is indicated by the red trace.

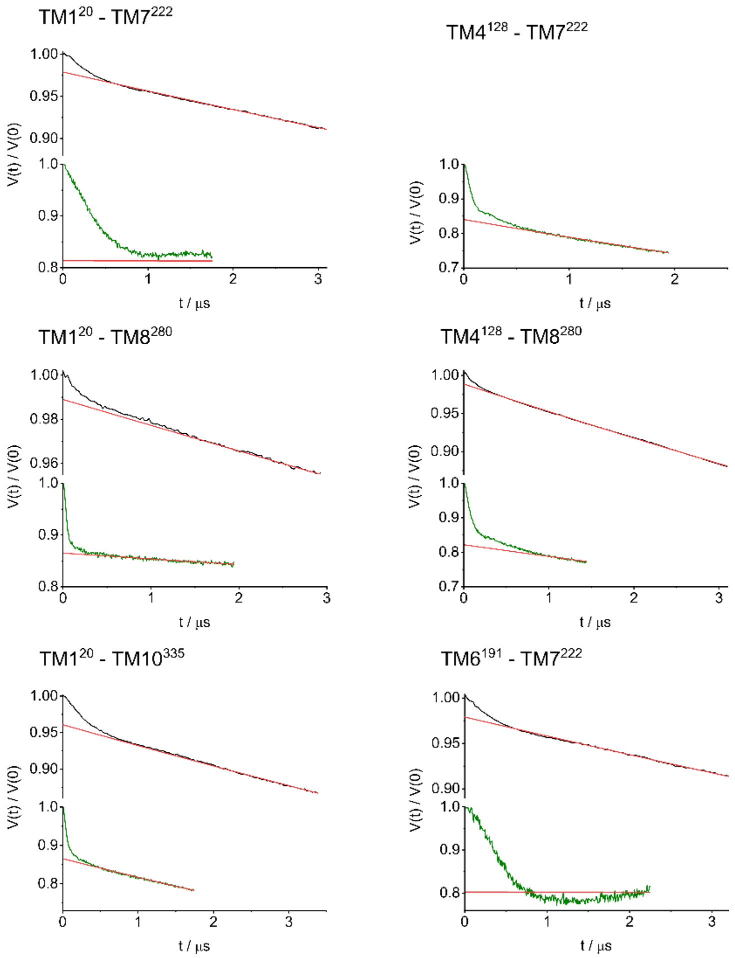

Figure S5: Raw data of all DEER experiments of cytoplasmic constructs shown in Fig. 5. The top panel of each dataset shows Gd(III) data (black trace) and the bottom panel MTSSL data (green trace). The background decay obtained by the DD software is indicated by the red trace.

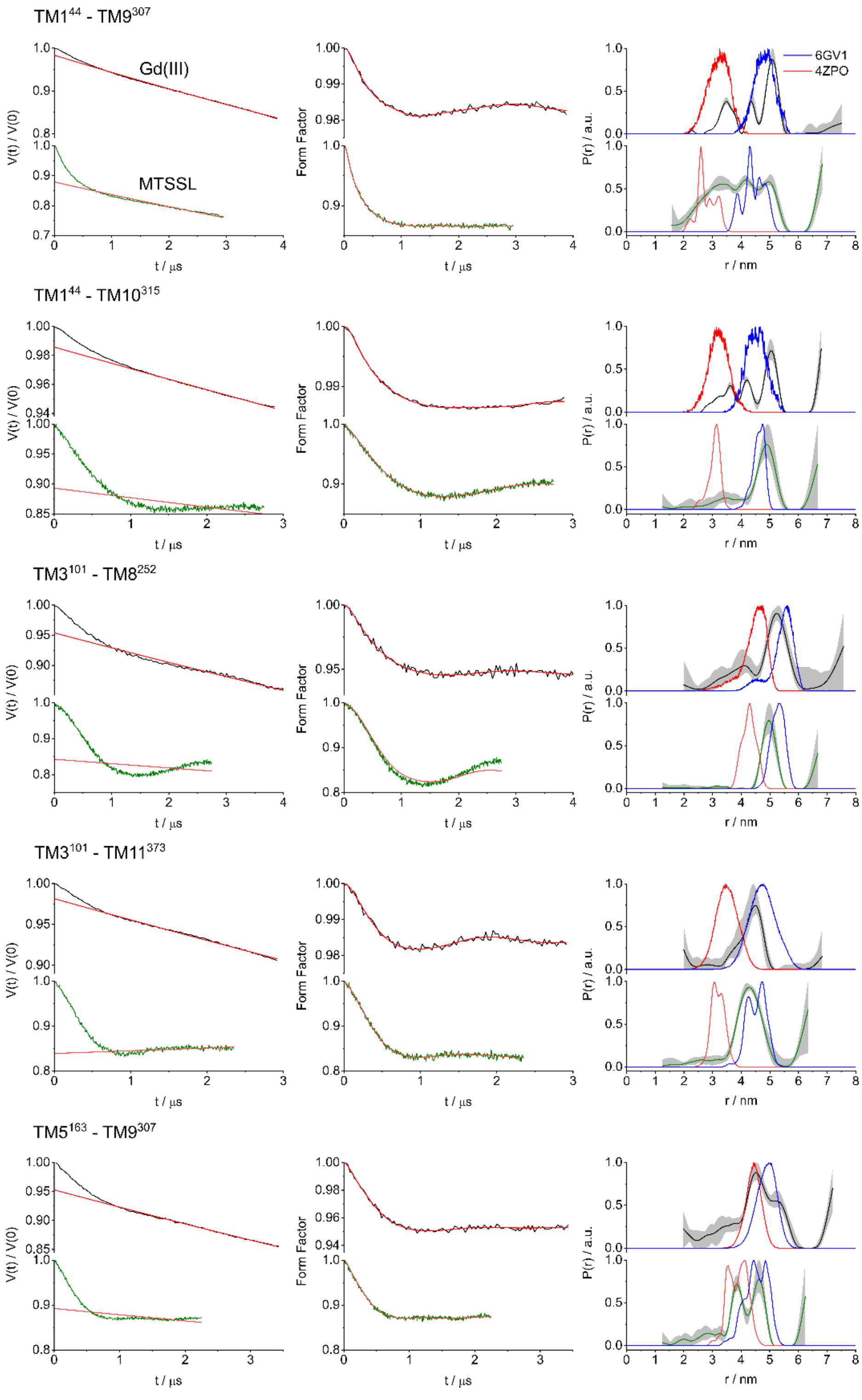

TM1 44 - TM9 307
TM1 44 - TM10 315
TM3 101 - TM8 252
TM3 101 - TM11 373
TM5 163 - TM9 307
Gd(III)
MTSSL
6GV1
4ZPO
V(t) / V(0)
Form Factor
P(r) / a.u.
t / μs
r / nm

(cont'd)

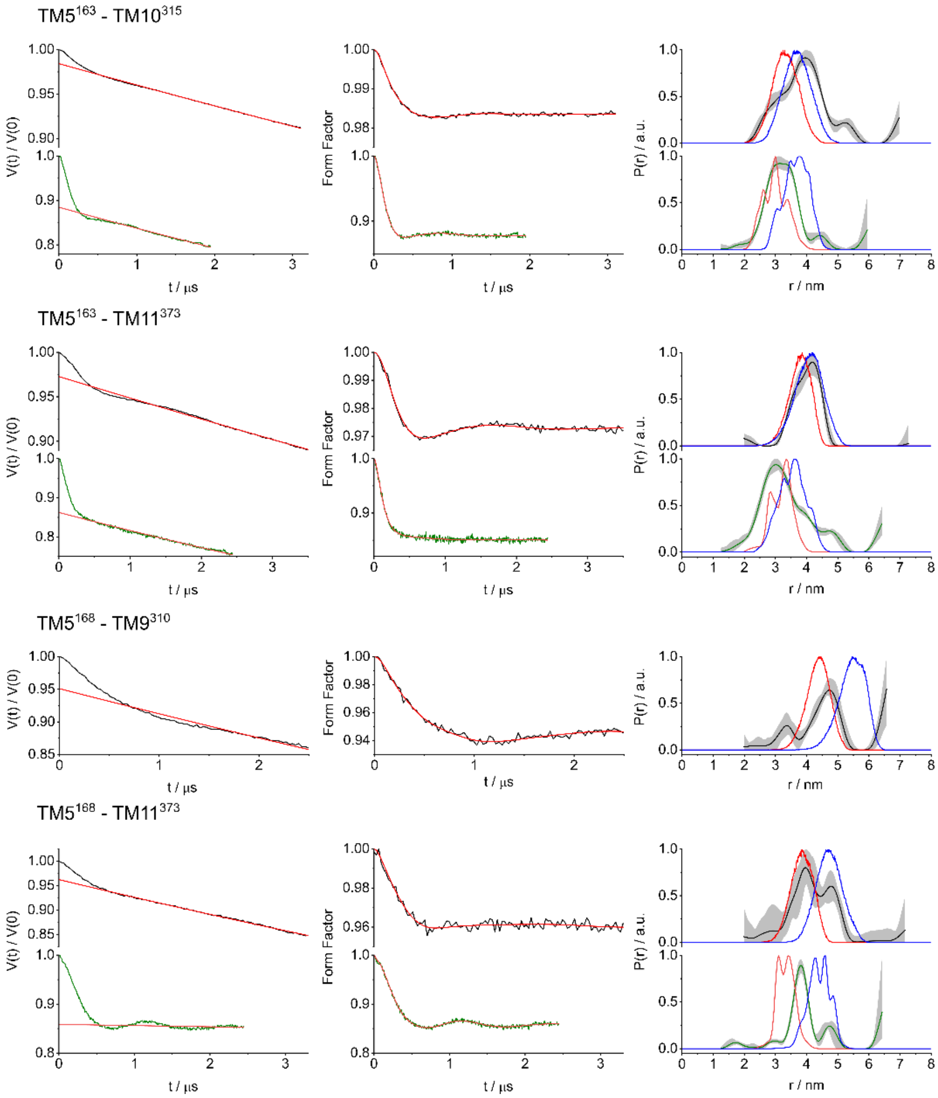

Figure S6: DEER data for all periplasmic mutants analyzed using Tikhonov regularization with DeerAnalysis 2018. The top panel of each dataset shows Gd(III) data (black trace) and the bottom panel MTSSL data (green trace, including the last 500 ns which were removed in Fig. S4 due to pulse overlapping). For every mutant, raw data with the background correction function (red, left column), form factor after background correction with the fit data (red) obtained with distance distribution on the right (middle column) and distance distribution (right column) are shown. The shaded gray area denotes the confidence bands for the distance distribution as obtained from the validation function in DeerAnalysis . The distance distribution calculated from the crystal structures 4ZP0 (I_f, red) and 6GV1 (O_o, blue) are shown as well.

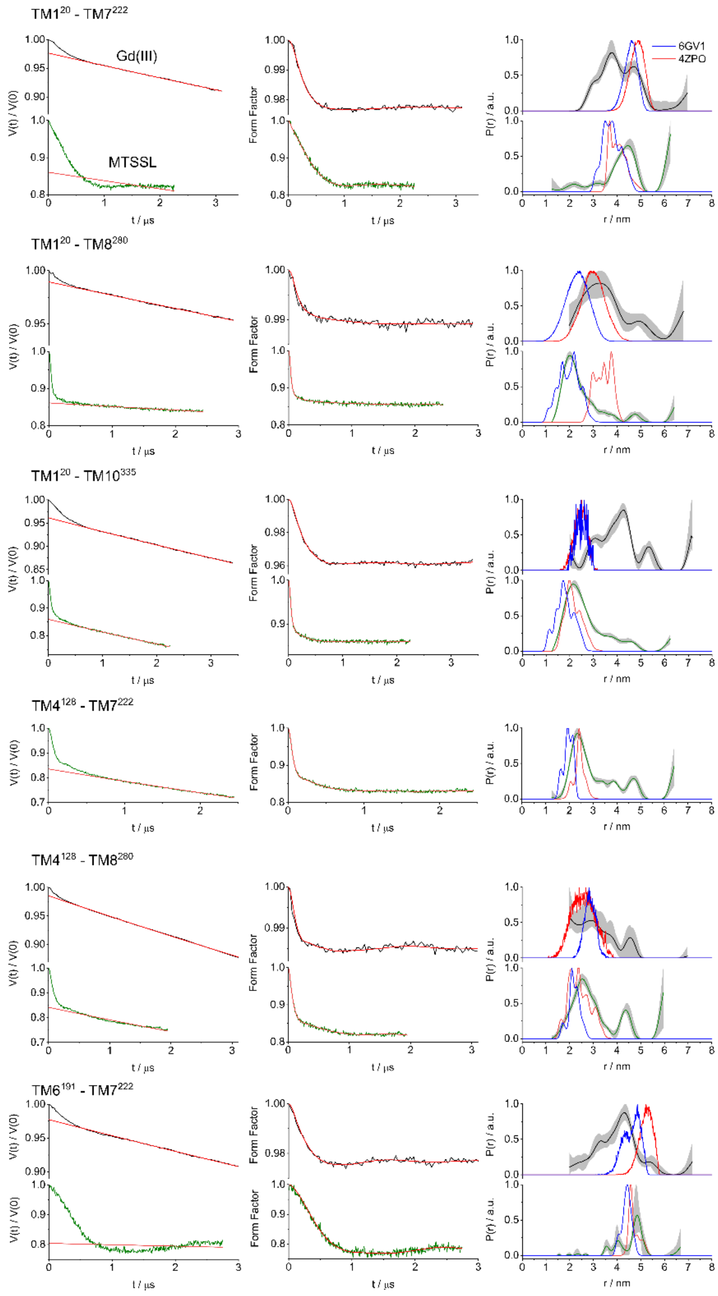

Figure S7: DEER data for all cytoplasmic mutants analyzed with Tikhonov regularization with DeerAnalysis 2018. The top panel of each dataset shows Gd(III) data (black trace) and the bottom panel MTSSL data (green trace, including the last 500 ns which were removed in Fig. S5 due to pulse overlapping). For every mutant, raw data with the background correction function (red, left column), form factor after background correction with the fit data (red) obtained with distance distribution on the right (middle column) and distance distribution (right column) are shown. The shaded gray area denotes the confidence bands for the distance distribution as obtained from the validation function in DeerAnalysis. The distance distribution calculated from the crystal structures 4ZP0 (I_f, red) and 6GV1 (O_o, blue) are shown as well.

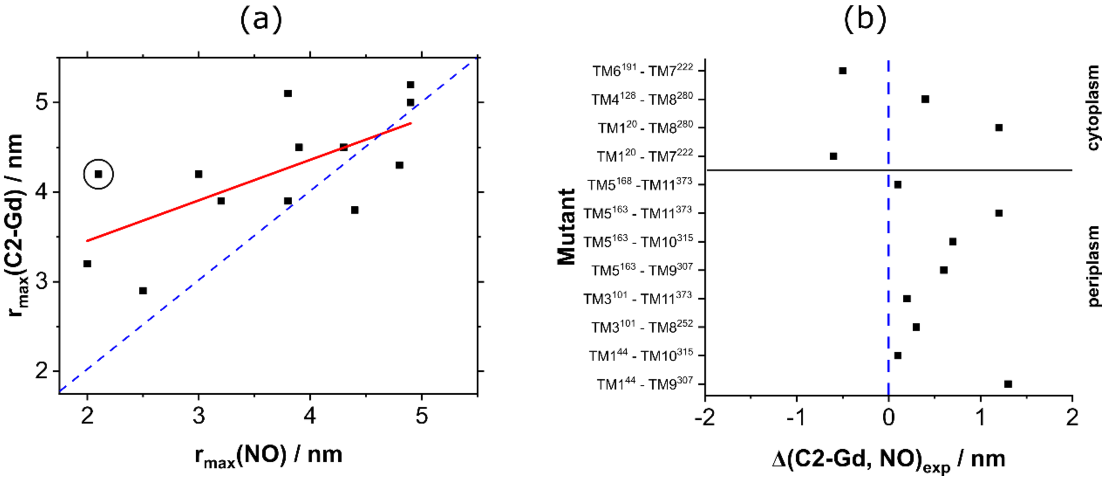

Figure S8: (a) Plot of the experimental r_{max} for the Gd-C2 labeled variants vs r_{max} for the NO labeled ones obtained from the analysis with Tikhonov regularization (see Fig.S6 and S7). When more than 1 peak appeared in the distance distribution, we considered only the major one. The blue, dotted line corresponds to a perfect match, whereas the red line is a linear fit. Mutant $TM1^{20}$ – $TM10^{335}$, which was removed from subsequent analysis as described in the text, is indicated by a black circle; (b) Plot of $\Delta(Gd,NO)_{exp}$ for each mutant studied, *except for* $TM1^{20}$ – $TM10^{335}$.

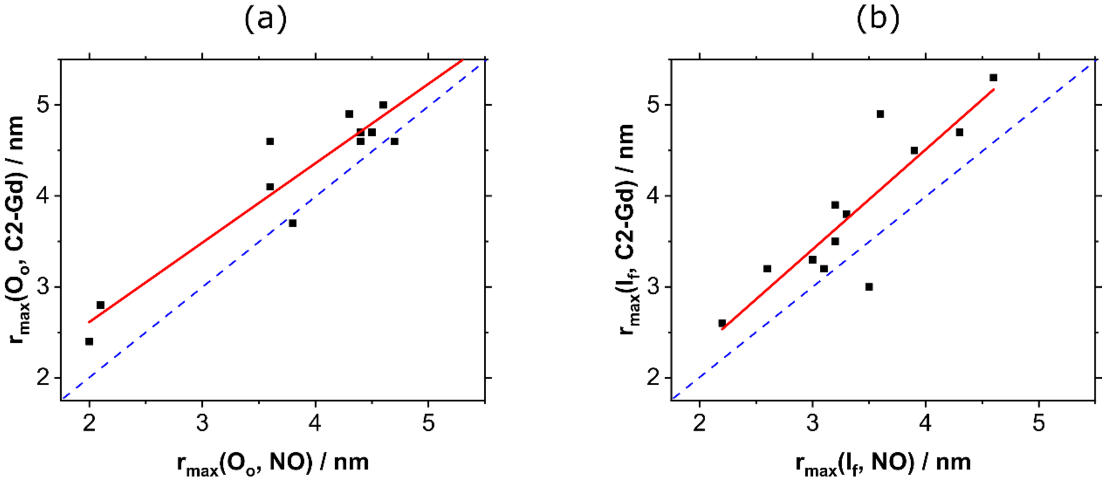

Figure S9: Plots of the calculated r_{max} (C2-Gd) vs r_{max} (NO) for the (a) O_o and (b) I_f structures. The dotted blue line corresponds to the perfect correlation and the red line is a linear fit.

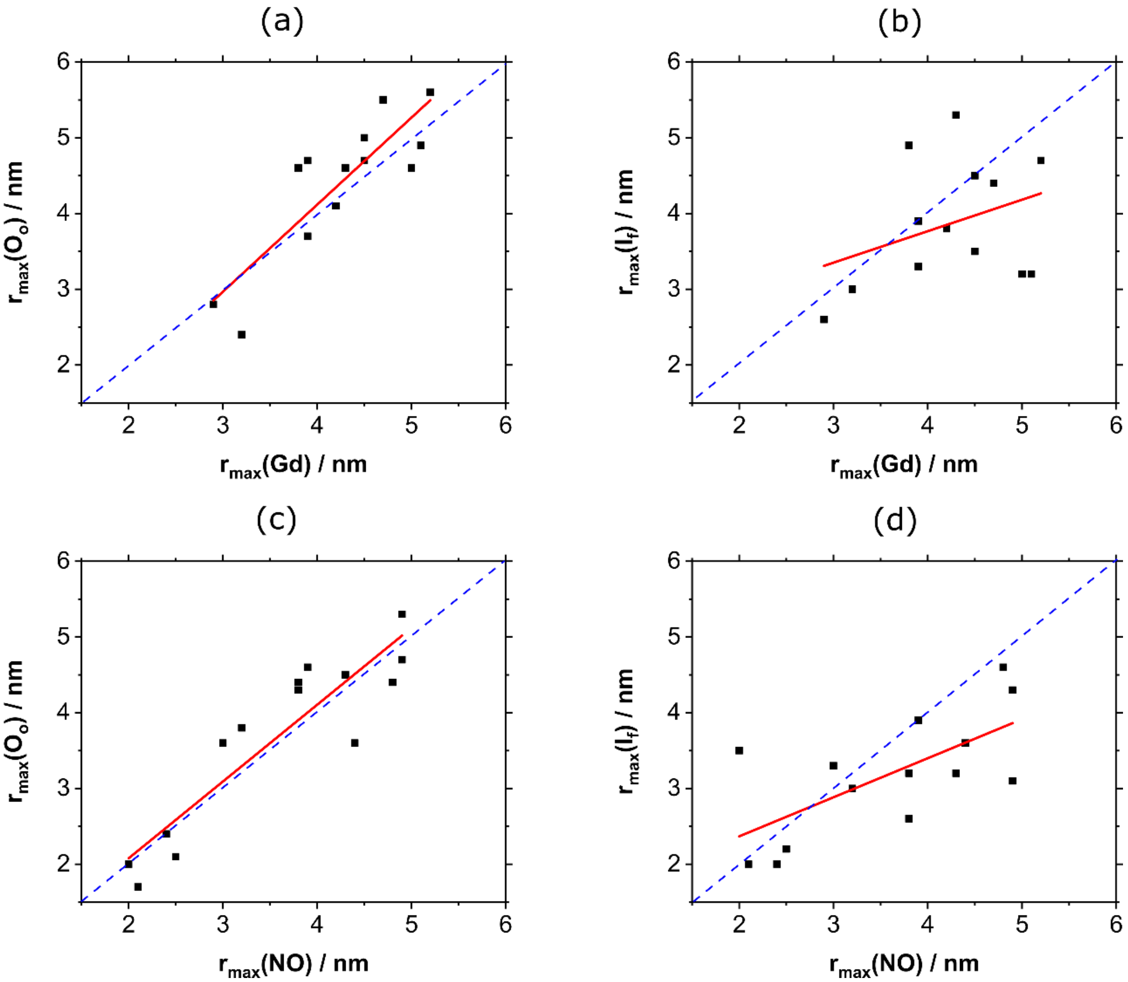

Figure S10: Comparison of the experimental and crystal structure derived r_{max} values for the Gd-C2 labelled mutants for the (a) 6GV1 (O_o) structure and (b) 4ZP0 (I_f) structure and the same for the NO labeled mutants (c,d). Experimental data were obtained using Tikhonov regularization. Blue dotted lines represent perfect correlation and the red line linear fits. Fit parameters were: slope = 1.15, R^2 = 0.73 (a), slope = 0.41, R^2 = 0.12 (b), slope = 1.01, R^2 = 0.83 (c), and slope = 0.51, R^2 = 0.46 (d).

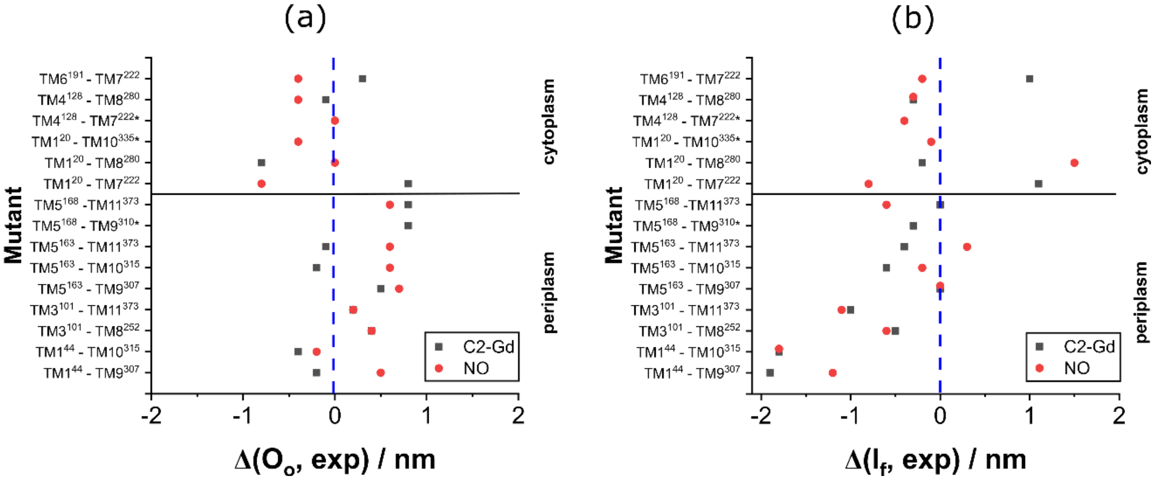

Figure S11: Plots showing the difference between the predicted and experimental r_{max} values calculated using Tikhonov regularization for the mutants studied (red symbols are for Gd-C2 and black for NO). (a) for the O_o and (b) I_f structure. The vertical blue dotted lines mark the zero lines (perfect agreement) and the black horizontal lines represent the division between periplasmic and cytoplasmic pairs.

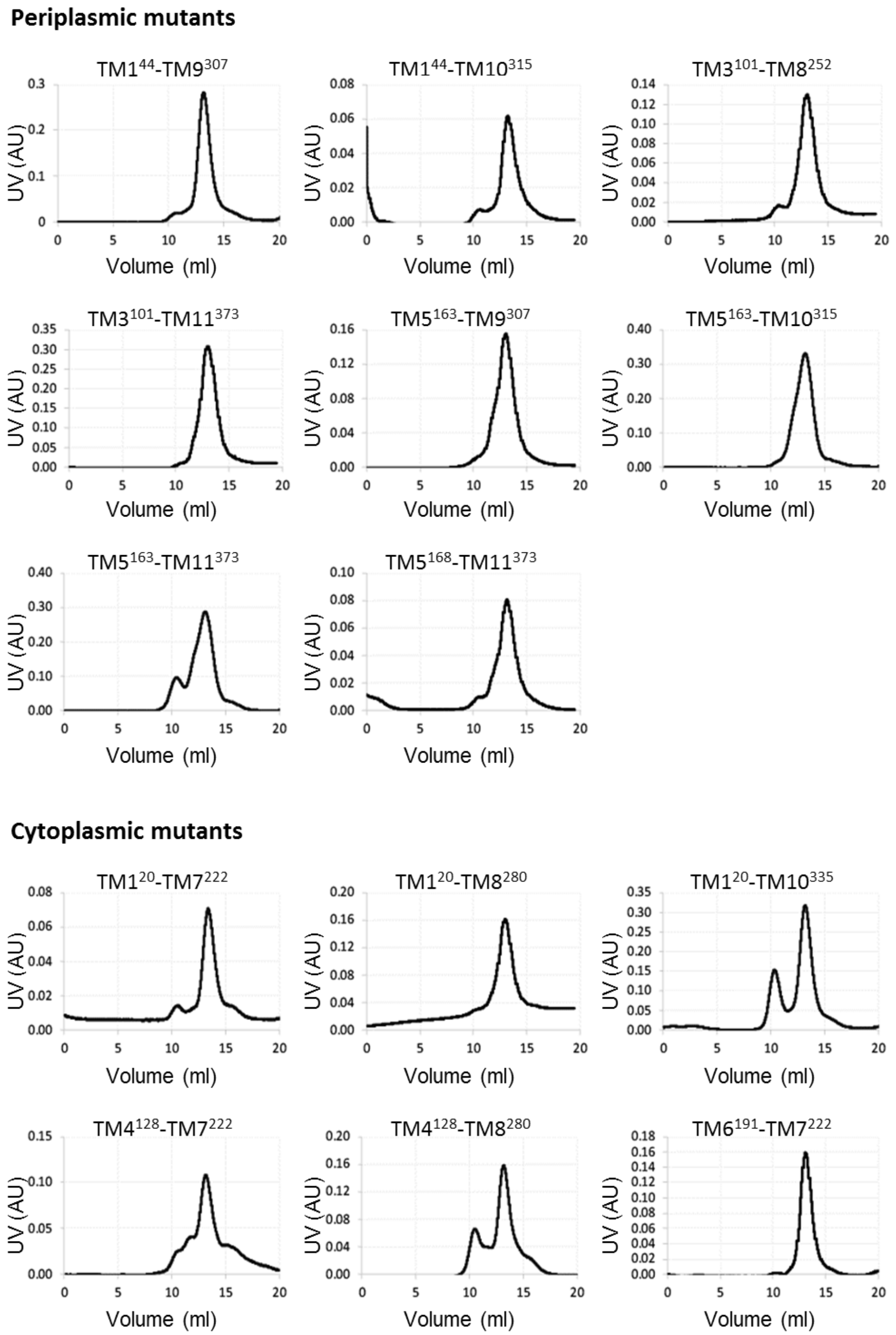

Figure S12: Size exclusion chromatography (SEC) elution profiles of all MTSSL labeled mutants. In cases where multiple peaks are visible, only the fractions in the major peak were used subsequently. In the TM1^{33} – TM10^{315} and TM6^{191} – TM7^{222} the UV detection was not zeroed.

Table S1: Overview of all mutants in the background of a cysteine-less MdfA, their designation in the text as well as the main distance r_{max} and (where applicable) the distance maximum of a second distance (in parenthesis) present in the data. Evaluation was performed using the software DD.

Mutant	Designation	r_{max} (NO)	r_{max} (Gd)
V44C W307C	$TM1^{44}$ - $TM9^{307}$	4.4 (3.2)	5.0 (3.5)
V44C W315C	$TM1^{44}$ - $TM10^{315}$	5.1	5.0 (4.1)
L101C L252C	$TM3^{101}$ - $TM8^{252}$	5.2	5.0
L101C L373C	$TM3^{101}$ - $TM11^{373}$	4.3	4.5 (3.6)
A163C V307C	$TM5^{163}$ - $TM9^{307}$	4.1	4.5
A163C W315C	$TM5^{163}$ - $TM10^{315}$	3.1	4.0 (3.0)
A163C L373C	$TM5^{163}$ - $TM11^{373}$	3.0	4.2 (3.6)
L168C S310C	$TM5^{168}$ - $TM9^{310}$	--	4.6
L168C L373C	$TM5^{168}$ - $TM11^{373}$	3.8	4.2
L20C V222C	$TM1^{20}$ - $TM7^{222}$	4.2	4.0
L20C S280C	$TM1^{20}$ - $TM8^{280}$	2.0	3.3
L20C V335C	$TM1^{20}$ - $TM10^{335}$	2.0 (2.4)	4.3 (3.4)
A128C V222C	$TM4^{128}$ - $TM7^{222}$	2.3 (3.9)	--
A128C S280C	$TM4^{128}$ - $TM8^{280}$	2.6 (4.5)	3.2
A191C V222C	$TM6^{191}$ - $TM7^{222}$	4.7	4.3 (3.6)

Table S2: Experimental conditions for all Gd(III)-Gd(III) DEER experiments. "AWG DEER" relates to a standard DEER experiment where the pump pulse is replaced by a linear chirp; "rDEER" indicates that the pump pulse is swept between the primary echo and the first π pulse. For details, refer to the experimental section in the main text. t_p and f_p are the pump pulse length and frequency, T indicates the time up to which the DEER trace was accumulated. Experiment run-time is given including hardware overhead.

Mutant	Experiment	t_p	f_p	T	Run-time
L20C V222C	rDEER	2 x 96 ns	94.55 - 94.85, 95.05 - 95.35	3.5 μs	9:30 h
L20C S280C	rDEER	2 x 96 ns	94.55 - 94.85, 95.05 - 95.35	3 μs	10:00 h
L20C V335C	rDEER	2 x 96 ns	94.55 - 94.85, 95.05 - 95.35	3.5 μs	3:20 h
V44C V307C	rDEER	2 x 96 ns	94.55 - 94.85, 95.05 - 95.35	4 μs	1:40 h
V44C W315C	rDEER	2 x 96 ns	94.55 - 94.85, 95.05 - 95.35	3 μs	5:30 h
L101C L252C	AWG DEER	2 x 76 ns	94.55 - 94.85, 95.05 - 95.35	3 μs	1:00 h
L101C L373C	rDEER	2 x 96 ns	94.55 - 94.85, 95.05 - 95.35	3 μs	3:00 h
A128C S280C	rDEER	2 x 96 ns	94.55 - 94.85, 95.05 - 95.35	3.2 μs	10:20 h
A163C V307C	AWG DEER	2 x 96 ns	94.55 - 94.85, 95.05 - 95.35	4 μs	5:40 h
A163C W315C	rDEER	2 x 96 ns	94.55 - 94.85, 95.05 - 95.35	3.2 μs	11:00 h
A163C L373C	rDEER	2 x 96 ns	94.55 - 94.85, 95.05 - 95.35	3.6 μs	7:30 h
L168C S310C	AWG DEER	2 x 96 ns	94.55 - 94.85, 95.05 - 95.35	3.5 μs	2:10 h
L168C L373C	AWG DEER	2 x 96 ns	94.55 - 94.85, 95.05 - 95.35	4 μs	1:10 h
A191C V222C	AWG DEER	2 x 96 ns	94.55 - 94.85, 95.05 - 95.35	3.5 μs	8:00 h
